# Deep Learning Networks Accurately Detect ST-Segment Elevation Myocardial Infarction and Culprit Vessel

**DOI:** 10.3389/fcvm.2022.797207

**Published:** 2022-03-10

**Authors:** Lin Wu, Guifang Huang, Xianguan Yu, Minzhong Ye, Lu Liu, Yesheng Ling, Xiangyu Liu, Dinghui Liu, Bin Zhou, Yong Liu, Jianrui Zheng, Suzhen Liang, Rui Pu, Xuemin He, Yanming Chen, Lanqing Han, Xiaoxian Qian

**Affiliations:** ^1^Department of Cardiology, The Third Affiliated Hospital, Sun Yat-sen University, Guangzhou, China; ^2^Department of Endocrine and Metabolic Diseases, The Third Affiliated Hospital, Sun Yat-sen University, Guangzhou, China; ^3^Center for Artificial Intelligence, Research Institute of Tsinghua, Pearl River Delta, Guangzhou, China; ^4^Novelty-Checking Center, Guangdong Institute of Scientific and Technical Information, Guangzhou, China; ^5^Department of Anesthesiology, The Third Affiliated Hospital, Sun Yat-sen University, Guangzhou, China; ^6^School of Computer Science and Engineering, Sun Yat-sen University, Guangzhou, China

**Keywords:** ST-segment elevation myocardial infarction (STEMI), electrocardiogram (ECG), convolutional neural network (CNN), long short-term memory (LSTM), CNN-LSTM, deep learning (DL), culprit vessel

## Abstract

Early diagnosis of acute ST-segment elevation myocardial infarction (STEMI) and early determination of the culprit vessel are associated with a better clinical outcome. We developed three deep learning (DL) models for detecting STEMIs and culprit vessels based on 12-lead electrocardiography (ECG) and compared them with conclusions of experienced doctors, including cardiologists, emergency physicians, and internists. After screening the coronary angiography (CAG) results, 883 cases (506 control and 377 STEMI) from internal and external datasets were enrolled for testing DL models. Convolutional neural network-long short-term memory (CNN-LSTM) (AUC: 0.99) performed better than CNN, LSTM, and doctors in detecting STEMI. Deep learning models (AUC: 0.96) performed similarly to experienced cardiologists and emergency physicians in discriminating the left anterior descending (LAD) artery. Regarding distinguishing RCA from LCX, DL models were comparable to doctors (AUC: 0.81). In summary, we developed ECG-based DL diagnosis systems to detect STEMI and predict culprit vessel occlusion, thus enhancing the accuracy and effectiveness of STEMI diagnosis.

## 1. Introduction

ST-segment elevation myocardial infarction (STEMI) is one of the leading cardiovascular diseases, with a high morbidity and mortality (1). ST-segment elevation is considered to reflect acute transmural ischemia caused by epicardial coronary artery blockage. The timely diagnosis of STEMI is crucial to guide therapy and lower sudden cardiac death (2). Coronary angiography (CAG) is the gold standard for diagnosing STEMI. However, CAG is an invasive, inconvenient, expensive, and radioactive examination and requires a monitoring infrastructure. As a result, an effective technique for screening STEMI patients is urgently required to distinguish STEMI from other diseases.

Twelve-lead electrocardiography (ECG) may potentially be a rapid, cost-effective, and non-invasive method of screening STEMI. However, it remains challenging to distinguish STEMI from other diseases with chest pain symptoms such as pneumothorax, aortic dissection, and pulmonary embolism (3). However, ST-segment elevation secondary to non-ischemic etiologies is widespread, which concurrently complicates the differential diagnosis of STEMI. Moreover, ischemic ST elevation is highly dynamic and demonstrates spontaneous elevation, such as Prinzmetal's variant angina pectoris. In addition, lead positioning changes, improper high-pass filter setting, QRS width, and axis may affect the magnitude of ST elevation. On the other hand, patients with real transmural ischemia are difficult to differentiate when their ST elevation was less than the recommended threshold. As a consequence, the specificity and sensitivity of ECG for STEMI are low (4).

Previous studies have used artificial intelligence (AI) networks to improve signal analysis, automated feature extraction, and non-linear models (5–7). Deep learning (DL)-based ECG interpretation focuses on noise reduction (8), ECG feature extraction (9), arrhythmia detection (10), and cardiovascular diseases (11). However, the application of this research on ECG has been limited by the small number of studies that employed data from the MIT-BIH (PhysioNET) and PTB (Physiobank) databases (7, 12). Moreover, several researchers employed the typical STEMI ECG pattern and excluded other ECG-related ST-segment changes, such as ventricular premature beat, left ventricular hypertrophy, complete left bundle branch block, and complete right bundle branch block. Salah Al-Zaiti et al. improved a machine learning model to predict ACS based on prospective real-world prehospital ECG data. However, CAG corroborated only part of this study's findings (13). In conclusion, certain models were machine learning-based, requiring extraction of the ECG features and then feeding them into the prediction model, while other models used DL to make end-to-end predictions, which only predicted two categories (control and STEMI).

To avoid the shortcomings of previous DL models, we aimed to develop highly effective and accurate DL models for diagnosing STEMI and culprit vessels. First, we established a real-world ECG dataset based on CAG. Then, we developed convolutional neural network (CNN), long short-term memory (LSTM), and CNN-LSTM models and investigated the accuracy and effectiveness of ECG-based STEMI detection.

## 2. Methods

### 2.1. Data Sources and Study Population

#### 2.1.1. Study Design

**Figure 1:**
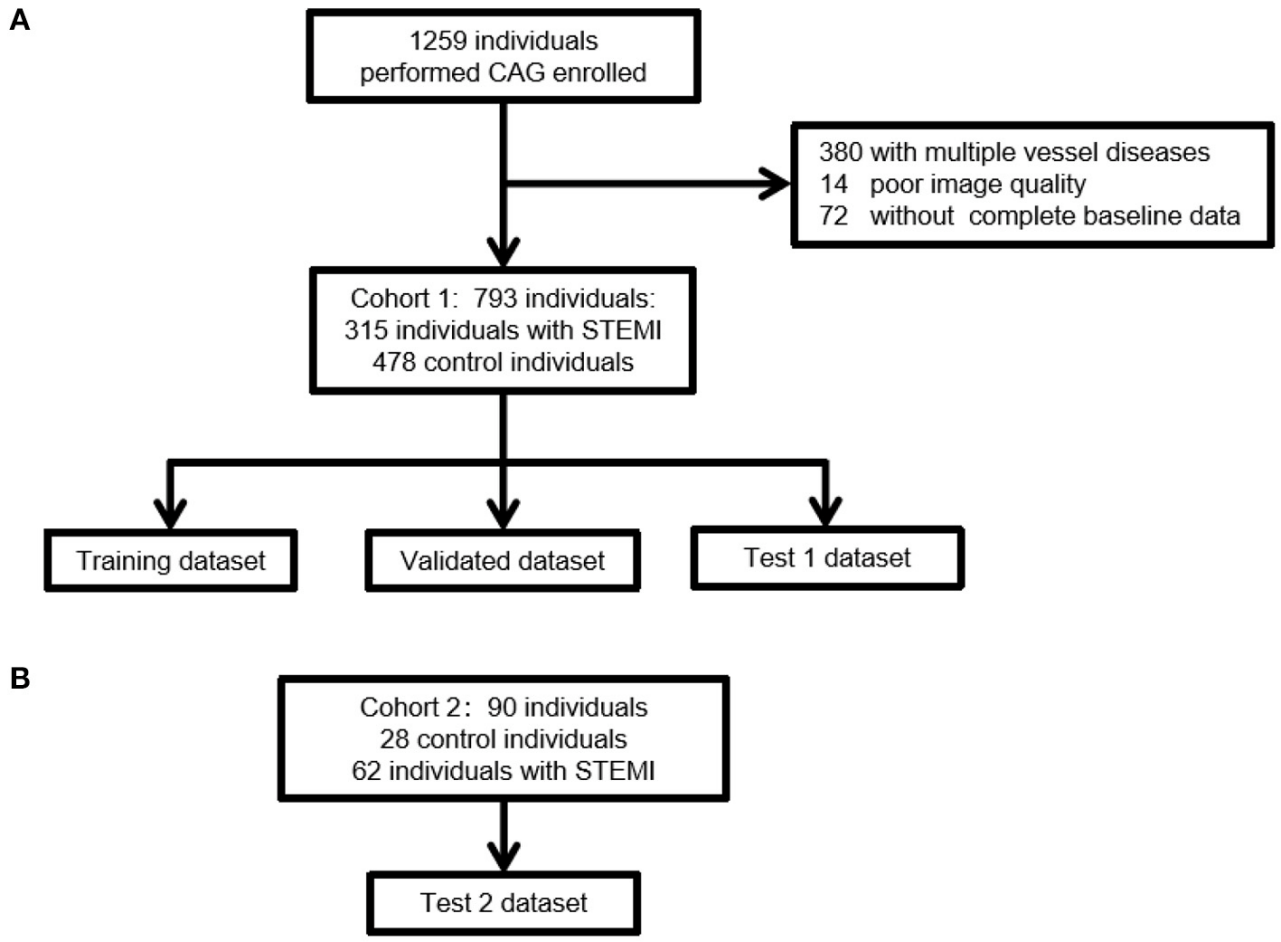
A flow diagram indicating the selection of individuals for the training, validation, and testing datasets. **(A)** The selection steps of Cohort 1 for the internal dataset. Of the 793 ECGs collected, Cohort 1 included 315 individuals with STEMI and 478 control individuals without STEMI. **(B)** The selection steps of external validation of ECG. Of the 90 ECGs collected, Cohort 2 included 62 individuals with STEMI and 28 control individuals without STEMI. The inclusion and exclusion criteria were consistent with those in Cohort 1. STEMI, ST segment elevation myocardial infarction.

This was a retrospective case-control study. To develop a training algorithm model, we selected STEMI and control ECG data from the Hospital Information System (HIS) and the Cardio-Catheter Room database from January 2015 to December 2018 in the Third Affiliated Hospital of Sun Yat-sen University (Cohort 1). The flowchart of the study is depicted in Figure 1. The inclusion criteria of STEMI ECG were as follows: final diagnosis of STEMI, without history of myocardial infarction or percutaneous coronary intervention (PCI). The inclusion criteria of control ECG were as follows: final diagnosis of STEMI, without history of myocardial infarction or PCI. The exclusion criteria included: Patients who need CAG for any reason do not reach the diagnosis of STEMI. Excessive noise in the data, unstable baseline, multiple vessel disease, no CAG performed during the first 24 h of the onset of symptoms (such as angina pain, chest pain, backache, shoulder pain, and stomachache), and no complete baseline data. The external test of ECG data was retrospectively collected from the ECG database of Guangzhou First People's Hospital (Cohort 2) (Figure 1B). The inclusion and exclusion criteria were consistent with those in Cohort 1. The diagnosis of myocardial infarction was based on clinical symptoms (angina pain lasting for over 20 min), ECG findings, and enzymatic changes. A cardiologist committee was composed of two board-certified practicing cardiac electrophysiologists and one board-certified practicing cardiologist. The committee discussed all ECG records and agreed on a gold standard of arrhythmia. The definitions of ST elevation at J points are based on the American College of Cardiology/American Heart Association and the European Society of Cardiology STEMI guidelines. This study was approved by the Human Ethics Boards of the Third Affiliated Hospital of Sun Yat-sen University and Guangzhou First People's Hospital. All of the studies were conducted in accordance with the Declaration of Helsinki. Informed consent was not required, as this was a retrospective study.

#### 2.1.2. ECG Data

A resting surface ECG was digital, standard, 10-s, 12-lead ECG. Electrocardiography was performed at a sampling rate of 1,000 Hz using a hospital ECG management system (ECGNET Vision 3.0, SanRui Electronic Technology, Guangdong, China). The raw ECG sequence data were acquired by a physician in the supine position (paper speed: 25 mm/s, calibration: 1 mV = 10 mm). All ECG data were labeled with the study ID and stored on a secure server. Initially, ECGs with excessive noise were excluded by two independent clinicians. The interpretation of ECG characteristics is shown in Supplementary Table 1. Diagnostic ST elevation is defined by the following Fourth Universal Definition of Myocardial Infarction consensus statement: (1) ST elevation in V2-V3 ≥ 2.0 mm in men ≥ 40 years, ≥ 2.5 mm in men < 40 years, or ≥ 1.5 mm in women, or ST-elevation ≥ 1 mm in other leads; (2) ST depression ≥ 0.5 mm; or (3) T-wave inversion ≥ 1 mm in leads with prominent R wave or R/S ratio ≥ 1.

### 2.2. Data Preprocessing

Electrocardiography data were stored by XML and parsed by the parser. We applied wavelet transform to remove noise. During signal processing, there was an apparent contrast between the wavelet component amplitude and noise component at high frequencies. After wavelet decomposition, wavelet coefficients with large amplitudes were mostly useful signals, while coefficients with small amplitudes were generally noise. To standardize the data, we utilized a 5-s segment ECG input model. The ECG data had 12 channels, and the specification of each segment of ECG data was finally intercepted (5,000, 12).

### 2.3. Model Development

**Figure 2:**
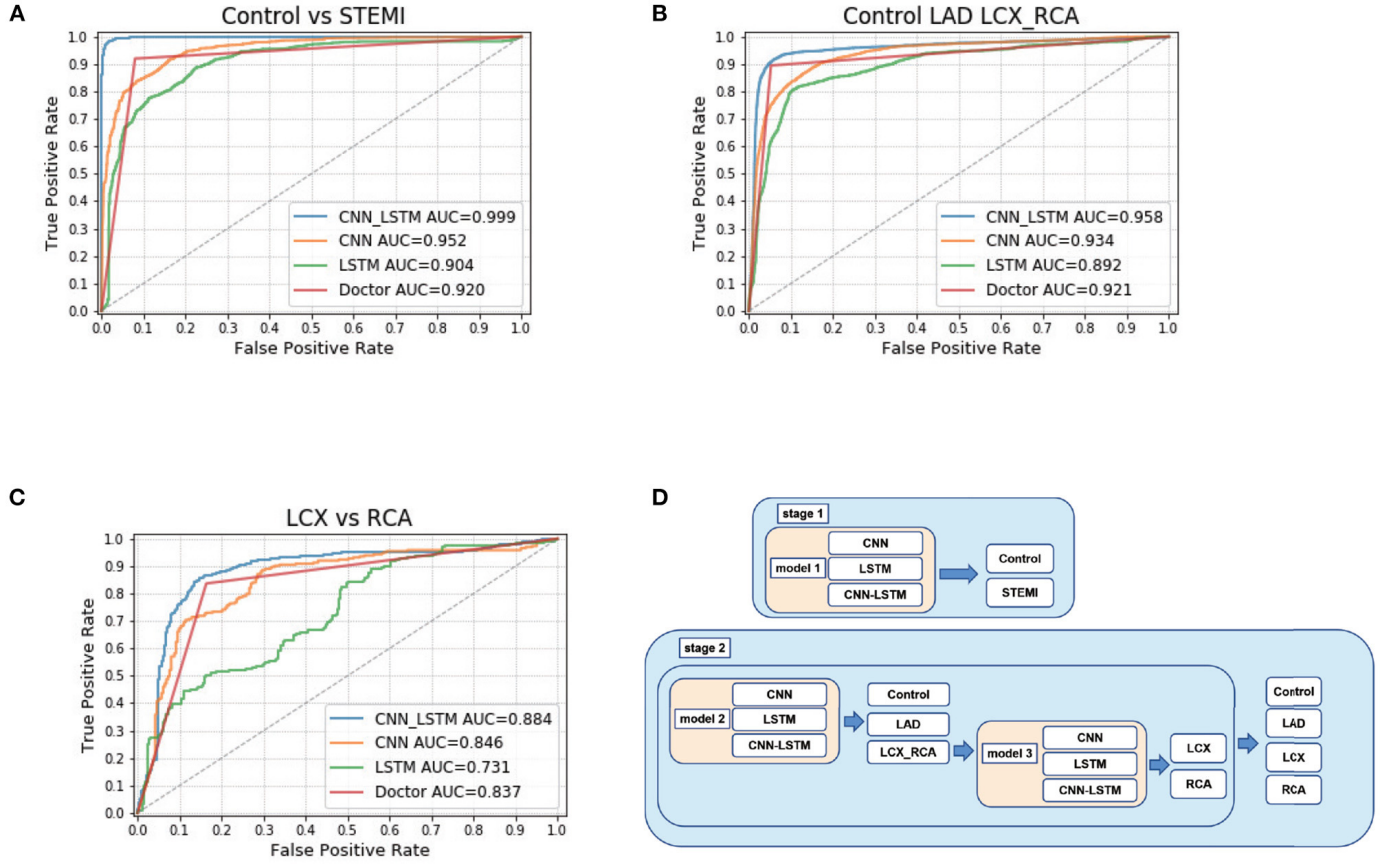
CNN-LSTM achieved the highest accuracy among CNN, LSTM, CNN-LSTM, and doctors. **(A)** ROC curve calculated at the sequence level for control and STEMI in the Test 2 dataset. **(B)** ROC curve calculated at the sequence level for control, LAD, LCX, and RCA. **(C)** ROC curve calculated for LCX and RCA. **(D)** Schematic of deep learning architecture.

In this study, we designed three different architectures (CNN, LSTM, and CNN-LSTM) to explore the best architecture of the model. Briefly, we designed two stages to classify different levels of STEMI (Figure 2D). The first stage (stage 1) was to train one model to distinguish between control and STEMI. The second stage (stage 2) was to train two models separately and then combine them to identify the control, LAD, LCX, and RCA. The first model in stage 2 identified the control, LAD, and LCX-RCA, whereas the second model in stage 2 identified the LCX and RCA.

#### 2.3.1. CNN

The CNN included three layers (Supplementary Materials and Supplementary Figure 1). Each kernel size was 2, and the number of kernels was randomly selected from a list of parameters 16, 24, 32, 48, and 64. Finally, the parameters of 32, 32, and 48 were the best for the CNN model to classify control, LAD, and LCX-RCA. The shape of input data was (5,000, 12) (Supplementary Figure 14). After the first CNN layer, the output data shape became (5,000, 32). Each CNN layer was followed by a pooling layer. After a pooling layer, the output data's shape was 2,500, 32. Similarly, the data shapes after the following layers were (2,500, 32), (1,250, 32), (1,250, 48), and (625, 48). Then, we added a dropout layer to increase the model's generalizability. Finally, we used a fully connected/dense layer with three nodes to predict the control, LAD, and LCX-RCA.

#### 2.3.2. LSTM

We built an LSTM model including two LSTM layers (Supplementary Materials and Supplementary Figure 2). The first LSTM layer possessed 100 neurons, and the second LSTM layer had 50 neurons. The numbers of LSTM neurons were randomly selected from a list of parameters of 500, 200, 100, and 50. The shape of input data was (5,000, 12) (Supplementary Figure 15). After the first LSTM layer, the output data's shape was (5,000, 100). After the second LSTM layer, the output data's shape was (50, 12). Each LSTM layer was followed by a dropout layer to enhance the model's generalization ability. Each dropout layer's dropout rate was 0.2. Finally, we utilized a fully connected/dense layer with two nodes to predict control and STEMI.

#### 2.3.3. CNN-LSTM

**Figure 3:**
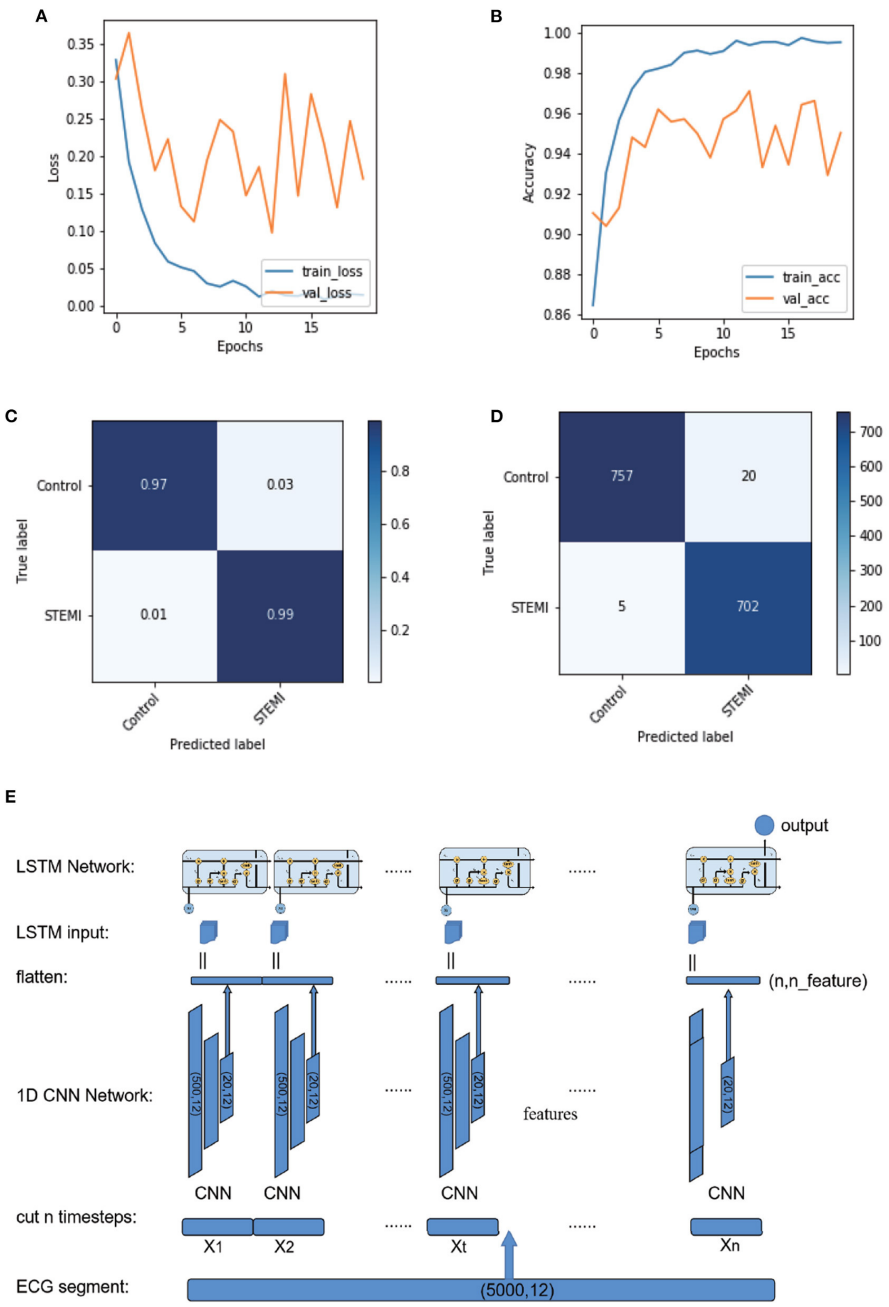
Plot of logarithmic accuracy/loss for the training step of CNN-LSTM. Train and test accuracy/loss diagrams of the models. **(A,B)** Train and test accuracy/loss diagrams of the optimum result on the CNN-LSMT model. **(C,D)** Confusion matrices for predicting control and STEMI using the CNN-LSTM model in the test dataset. **(E)** Schematic of the CNN-LSTM architecture.

We established a CNN-LSTM model that combined CNN and LSTM (Figures 3C–E; Supplementary Materials and Supplementary Figure 2). The final output was (50, 12, and 48), reshaped into (50, 576), and then fed into subsequent LSTM networks (Supplementary Figure 16). We set 50 LSTM cells to calculate 50 samples of 576 dimensions, which became 50 dimensions after calculation (Supplementary Figure 16). The last layer of the model was a fully connected layer, with two nodes set as outputs, corresponding to the probability of control and STEMI (Figures 3A,B).

### 2.4. Comparative Test

Analysis of ECG images was performed by 16 doctors for comparison with our DL algorithms. Each doctor made a diagnosis blindly and independently. A total of 16 doctors contained four medical interns, four internal medicine residents and four experienced cardiologists, and four emergency physicians. The medical interns had completed the theoretical studies of cardiology and ECG. The internal medicine residents were doctors who held a medical license but had not majored in cardiology. The experienced cardiologists had at least 5-year experience working in cardiovascular department. The emergency physicians had at least 2-year experience working in emergency department. Each doctor made a diagnosis blindly and independently.

### 2.5. Statistical Analysis

Baseline characteristics in the STEMI and control groups were retrieved using inpatient ICD-9 and ICD-10 diagnostic codes. Continuous data are expressed as the mean value ± standard deviation, and categorical data are presented as absolute numbers and percentages. Differences in continuous data were analyzed using Student's *t*-test for variables with a normal distribution and the Mann-Whitney U-test for variables with a non-normal distribution. Categorical data were analyzed using Pearson's chi-square test. The different performance metrics were AUC, sensitivity, specificity, positive predictive value (PPV), and negative predictive value (NPV) evaluated for a certain classification cut off value. *P* < 0.05 was considered statistically significant. IBM SPSS version 26.0 software was used for all statistical analyses.

## 3. Results

### 3.1. Study Population and Clinical Characteristics

A total of 1,259 individuals performed CAG were collected in the Third Affiliated Hospital of Sun Yat-sen University (Figure 1A). Patients had multiple vessel diseases or with poor image quality or without complete baseline data were excluded. Finally, 793 patients were enrolled in Cohort 1. In this cohort, 315 patients had STEMI, of which, 163 patients had isolated LAD artery branch disease, 107 patients had isolated right coronary artery (RCA), and 45 patients had isolated left circumflex artery (LCX). The control group included 478 patients without obstructive coronary artery disease. A total of 90 patients were enrolled from January 2017 to December 2018 in Guangzhou First People's Hospital. This cohort included 62 patients with STEMI and 28 control subjects. Thirty-one patients had isolated LAD branch disease, 21 patients had isolated RCA, and 10 patients had isolated LCX. Overall, STEMI patients were older than those without STEMI (control: 55.3±12.5; STEMI: 60.4±13.2, Table 1). There were significantly more males in the STEMI group than in the control group. No significant difference was observed in the prevalence of diabetes, chronic kidney disease, or family history of cardiovascular disease between the STEMI and control groups. Notably, the proportion of hypertension in STEMI patients was lower than that in the control group. No significant difference was identified in age, gender, prevalence of chronic kidney disease, or CVD family history between Cohorts 1 and 2. However, the prevalence of hypertension and diabetes was higher in Cohort 1 than in Cohort 2 (Table 2). In our study, we did not exclude arrhythmias. There were 18 ventricular premature beats, one complete left bundle branch block, five atrial fibrillation, and two ventricular preexcitation cases in the control and STEMI groups. In the control group, 19 left ventricular hypertrophy and nine complete right bundle branch block cases were identified, whereas the STEMI group included eight left ventricular hypertrophy, 11 complete right bundle branch block, and one pacing case. There was no statistically significant difference in the cardiac arrhythmia ratio (Table 1).

**Table 1:** Baseline characteristics between patients with or without STEMI.

	**Control**	**STEMI**	***P*-value**
	506	377	
Age (years)	55.3 ± 12.5	60.4 ± 13.2	0.000
Gender (female)	227 (44.9%)	69 (18.3%)	0.000
Diabetes mellitus	101 (20.0%)	71 (18.8%)	0.676
Hypertension	226 (44.7%)	135 (35.8%)	0.008
Chronic kidney disease	12 (2.4%)	9 (2.4%)	0.989
CVD family history	23 (4.5%)	21 (5.6%)	0.489
White blood cell (*10^9^/L)	7.33 ± 2.98	9.01 ± 3.12	0.000
Red blood cell (*10^9^/L)	4.28 ± 0.95	4.39 ± 0.69	0.039
Hemoglobin (g/ml)	128.05 ± 16.84	131.16 ± 19.52	0.011
Platelet (*10^9^/L)	207.21 ± 59.3	218.37 ± 61.25	0.007
ALB (g/L)	39.29 ± 4.43	38.61 ± 4.69	0.028
Globulin (g/L)	24.65 ± 3.91	25.18 ± 4.83	0.070
Potassium (mmol/L)	3.58 ± 0.42	3.78 ± 0.52	0.000
Sodium (mmol/L)	137.47 ± 4.61	137.91 ± 4.96	0.179
Ca (mmol/L)	2.15 ± 0.31	2.13 ± 1.16	0.697
Fasting glucose (mmol/L)	6.71 ± 2.99	7.17 ± 2.82	0.020
Blood urea nitrogen (mmol/L)	5.12 ± 2.74	5.77 ± 3.00	0.001
Serum creatinine (mmol/L)	81.71 ± 58.42	77.07 ± 44.97	0.199
CHOL (mmol/L)	4.52 ± 1.23	4.50 ± 1.22	0.860
TG (mmol/L)	1.59 ± 1.08	1.65 ± 1.12	0.461
HDL (mmol/L)	1.09 ± 0.32	1.00 ± 0.3	0.000
LDL (mmol/L)	2.79 ± 1.01	2.94 ± 1.04	0.042
CK-MB (U/L)	6.76 ± 6.31	32.83 ± 58.49	0.000
Ventricular premature beat	18 (3.6%)	18 (4.8%)	0.073
Preexcitation syndrome	2 (0.4%)	2 (0.5%)	0.617
Complete left bundle branch block	1 (0.2%)	1 (0.3%)	1.000
Complete right bundle branch block	9 (1.8%)	11 (2.9%)	0.094
Left ventricular hypertrophy	19 (3.8%)	8 (2.1%)	0.682
Atrial fibrillation	5 (1.0%)	2 (0.5%)	1.000
Pacing	0 (0)	1 (0.3%)	0.352

**Table 2:** Diagnostic performance of CNN-LSTM, CNN, LSTM, and doctors in different datasets.

	***n***	**AUC**	**ACC**	**SEN**	**SPEC**	**PPV**	**NPV**	**F1**
Model 1 Test 1								
CNN	1,393	0.95	0.87	0.90	0.91	0.83	0.84	0.87
LSTM	1,801	0.90	0.83	0.78	0.80	0.85	0.81	0.84
CNN-LSTM	1,484	1.00	0.98	0.97	0.97	0.99	0.99	0.98
Model 1 Test 2								
CNN	1,857	0.96	0.84	0.90	0.69	0.99	0.99	0.94
LSTM	1,857	0.95	0.86	0.93	0.79	0.94	0.92	0.93
CNN-LSTM	1,857	0.99	0.91	0.94	0.83	0.98	0.98	0.96
Model 2 Test 1								
CNN	3,395	0.93	0.79	0.78	0.89	0.81	0.89	0.80
LSTM	1,801	0.89	0.77	0.78	0.91	0.67	0.91	0.72
CNN-LSTM	3,395	0.96	0.89	0.83	0.91	0.9	0.91	0.87
Model 2 Test 2								
CNN	1,857	0.93	0.79	0.72	0.82	0.88	0.82	0.79
LSTM	1,857	0.89	0.79	0.86	0.94	0.74	0.94	0.79
CNN-LSTM	1,857	0.96	0.87	0.83	0.90	0.95	0.90	0.89
Model 3 Test 1								
CNN	263	0.70	0.70	0.68	0.68	0.93	0.93	0.79
LSTM	263	0.77	0.78	0.78	0.78	0.94	0.94	0.85
CNN-LSTM	263	0.81	0.67	0.66	0.66	0.92	0.92	0.77
Model 3 Test 2								
CNN	710	0.75	0.69	0.73	0.73	0.78	0.78	0.75
LSTM	710	0.70	0.68	0.64	0.64	0.83	0.83	0.73
CNN-LSTM	710	0.68	0.59	0.69	0.69	0.69	0.69	0.69

### 3.2. Model Performance

Misdiagnosis of STEMI may lead to severe events such as sudden death. Overdiagnosis of other diseases with ST-segment elevation would waste medical resources and needlessly invasive treatments. To accurately assess STEMI, we constructed CNN and LSTM models, combined them and developed a classifier to improve the accuracy of STEMI diagnosis.

#### 3.2.1. Stage 1: Control vs. STEMI

The first step was to distinguish between STEMI and control cases. First, after using a 10-fold cross-validation approach, we trained CNN, LSTM, and CNN-LSTM using each ECG raw data in the training dataset, followed by testing model performance in the internal test dataset (Test 1) and external test dataset (Test 2). Using the optimal probability threshold in CNN, the best AUC of STEMI was 0.95 (F1 score: 0.87) (Table 2; Figure 2A) in Test 1 and 0.96 (F1 score: 0.94) in Test 1 and Test 2, respectively. Second, after training, we validated the sensitivity, specificity, PPV, and NPV between the control and STEMI groups in two tests. The results of LSTM were 0.78, 0.80, 0.85, and 0.81 in the Test 1 dataset and 0.93, 0.79, 0.94, and 0.92 in the Test 2 dataset, respectively. Finally, we combined CNN and LSTM models to obtain a better performance with AUCs of 1.00 and 0.99 in Test 1 and Test 2, respectively (Figures 2C,D). The sensitivity, specificity, PPV, NPV, ACC, and F1 scores were improved after combining CNN and LSTM (Table 2; Figure 2A; Supplementary Figures 4–6).

#### 3.2.2. Stage 2 Model 1: Control vs. LAD vs. RCA-LCX

ROC analysis was utilized to discriminate the location of culprit vessels (Figure 2B). Interestingly, three models performed well in determining the location of culprit vessels, especially in the LAD subgroups (Table 2; Figure 2B). In Test 1 and Test 2, CNN achieved AUCs of 0.93 and 0.93, and LSTM achieved AUCs of 0.89 and 0.89, respectively. After combining CNN and LSTM as CNN-LSTM, the model performance improved to 0.96 and 0.97 in Test 1 and Test 2, respectively. The detailed analysis was summarized in Table 2. The accuracy and AUC of CNN-LSTM were better than those of CNN and LSTM in Tests 1 and 2 (Supplementary Figures 7–9).

#### 3.2.3. Stage 2 Model 2: RCA vs. LCX

Stage 2 model 2 was used to distinguish between RCA and LCX (Table 2; Figure 2C). The AUC values of CNN, LSTM, and CNN-LSTM in Test 1 were 0.70, 0.77, and 0.81, respectively, indicating that CNN and LSTM as single methods performed worse than CNN-LSTM. However, the F1 scores of CNN-LSTM in Tests 1 and 2 were worse than those of CNN and LSTM. However, the PPV and NPV of CNN-LSTM were similar to those of CNN and LSTM (Supplementary Figures 10–12).

### 3.3. Generalization Capacity

The CNN specificity differed significantly in model 1. For PPV and NPV, CNN performance in models 2 and 3 was inconsistent. The PPV and NPV of LSTM were unbalanced in models 1, 2, and 3. The generalization capacity of CNN-LSTM in the independent test dataset was better than that in CNN and LSTM (Table 3). For the model performance of CNN-LSTM in models 1 and 2, no significant difference was observed between Tests 1 and 2. For AUC, PPV, and NPV, the performance of CNN-LSTM was inconsistent in model 3 (Supplementary Figures 4–12).

**Table 3:** Diagnosis performance of CNN-LSTM, CNN, and LSTM between internal and external test.

		***n***	**AUC**	**ACC**	**SEN**	**SPEC**	**PPV**	**NPV**	**F1**
Model 1									
CNN-LSTM	Test 1	1,484	1.00	0.98	0.97	0.97	0.99	0.99	0.98
	Test 2	1,857	0.99	0.91	0.94	0.83	0.98	0.98	0.96
CNN	Test 1	1,393	0.95	0.87	0.90	0.91	0.83	0.84	0.87
	Test 2	1,857	0.96	0.84	0.90	0.69	0.99	0.99	0.94
LSTM	Test 1	1,801	0.90	0.83	0.78	0.80	0.85	0.81	0.84
	Test 2	1,857	0.95	0.86	0.93	0.79	0.94	0.92	0.93
Model 2									
CNN-LSTM	Test 1	1,484	1.00	0.98	0.97	0.97	0.99	0.99	0.98
	Test 2	1,857	0.99	0.91	0.94	0.83	0.98	0.98	0.96
CNN	Test 1	1,393	0.95	0.87	0.90	0.91	0.83	0.84	0.87
	Test 2	1,857	0.96	0.84	0.90	0.69	0.99	0.99	0.94
LSTM	Test 1	1,801	0.90	0.83	0.78	0.80	0.85	0.81	0.84
	Test 2	1,857	0.95	0.86	0.93	0.79	0.94	0.92	0.93
Model 3									
CNN-LSTM	Test 1	263	0.81	0.67	0.66	0.66	0.92	0.92	0.77
	Test 2	710	0.68	0.59	0.69	0.69	0.69	0.69	0.69
CNN	Test 1	263	0.70	0.70	0.68	0.68	0.93	0.93	0.79
	Test 2	710	0.75	0.69	0.73	0.73	0.78	0.78	0.75
LSTM	Test 1	263	0.77	0.78	0.78	0.78	0.94	0.94	0.85
	Test 2	710	0.70	0.68	0.64	0.64	0.83	0.83	0.73

### 3.4. Comparative Test

In the comparative test of model 1, CNN-LSTM achieved an AUC of 1.00, outperforming experienced cardiologists (0.94), emergency physicians (0.92), internal medicine residents (0.92), and medical interns (0.91). Doctors' performance at all levels was comparable in distinguishing STEMI from the control (Table 4; Figures 2A–C). In model 2, to determine LAD, there was no significant difference in predicting performance among CNN-LSTM, experienced cardiologists, and emergency physicians. In model 3, CNN-LSTM had the highest AUC (0.81) compared to doctors' performance at all levels. When comparing LCX to RCA cases, CNN-LSTM had a sensitivity and specificity of 0.70 and 0.70, respectively, which were slightly lower than those obtained from experienced cardiologists (sensitivity: 0.75; specificity: 0.75) and emergency physicians (sensitivity: 0.75; specificity: 0.75) (Supplementary Figure 13).

**Table 4:** Diagnosis performance of CNN-LSTM and of different levels of doctors.

	**Cases**	**AUC**	**ACC**	**SEN**	**SPEC**	**PPV**	**NPV**	**F1**
Model 1								
CNN-LSTM	78	1.00	1.00	1.00	1.00	1.00	1.00	1.00
DOCTOR	147	0.92	0.92	0.92	0.92	0.92	0.92	0.92
experienced cardiologists	147	0.94	0.94	0.94	0.94	0.94	0.94	0.93
emergency physicians	147	0.92	0.92	0.91	0.91	0.92	0.92	0.91
internal medicine residents	147	0.92	0.91	0.91	0.91	0.91	0.91	0.91
medical interns	147	0.90	0.90	0.90	0.90	0.90	0.90	0.90
Model 2								
CNN-LSTM	81	0.96	0.93	0.88	0.90	0.79	0.90	0.83
DOCTOR	147	0.92	0.90	0.85	0.94	0.87	0.94	0.89
experienced cardiologists	147	0.94	0.92	0.88	0.95	0.88	0.95	0.88
emergency physicians	147	0.93	0.93	0.86	0.94	0.89	0.94	0.87
internal medicine residents	147	0.91	0.88	0.81	0.92	0.84	0.92	0.82
medical interns	147	0.91	0.88	0.84	0.93	0.85	0.93	0.85
Model 3								
CNN-LSTM	14	0.81	0.71	0.70	0.70	0.88	0.88	0.78
DOCTOR	147	0.84	0.84	0.72	0.72	0.87	0.87	0.72
experienced cardiologists	147	0.86	0.86	0.75	0.75	0.89	0.89	0.78
emergency physicians	147	0.87	0.87	0.75	0.75	0.92	0.92	0.80
internal medicine residents	147	0.81	0.81	0.69	0.69	0.79	0.79	0.72
medical interns	147	0.82	0.82	0.68	0.68	0.86	0.86	0.70

## 4. Discussion

ST-segment elevation myocardial infarction is the most dangerous type of coronary heart disease, having the highest mortality and disability rates. It has been reported that inappropriate and false-positive activation of the cardiac catheterization laboratory for STEMI was approximately 2.7 % and 20 % (14, 15). However, due to the different levels of diagnosis and uneven coverage of medical resources, there is a large chance for misdiagnosis and missed diagnosis of STEMI, which affects the optimal diagnosis and treatment strategy. Electrocardiography is an objective, inexpensive, and widely used diagnostic tool for STEMI that provides guidance for revascularization. When a doctor performs an electrocardiogram diagnosis, the doctor often makes the diagnosis inconsistent due to different experiences, and it is time-consuming and labor-intensive. Based on ECG, the rapid diagnosis of STEMI using AI technology represents a significant progress in shortening the Door to Balloon time, as shorter Door to Balloon times were closely associated with lower in-hospital mortality and 6-month mortality (16).

### 4.1. Based on a Real-World ECG Database

We established a real-world ECG database containing arrhythmias that affect ST segment changes, including ventricular premature beat, ventricular preexcitation, pacemaker, bundle branch block, and so on. As far as we know, previous studies are primarily based on ECG data obtained from the MIT-BIH (PhysioNET) and PTB databases (Physiobank) (7, 12), both of which eliminated arrhythmia. By including arrhythmia data, our DL model achieves better sensitivity and specificity. Furthermore, we input the raw ECG data rather than extracting features from it. In this way, we are able to reduce feature loss, maintain data integrity, and improve diagnosis accuracy. Therefore, compared to previous models, our model is more realistic and is easier to apply in primary medical units.

### 4.2. Input the Raw ECG Data

Compared to previous ML or DL methods that require a very large amount of labeled data and time-consuming human labeling effort (17), our CNN-LSTM model is an end-to-end approach that does not require such efforts. It only utilized raw ECG data input and built binary classification and multiclassification without experts or experienced cardiologists. On the internal test dataset, our DL models achieved high sensitivity and NPV. A previous study reported that an ML-based ECG autodiagnostic system could improve the sensitivity of STEMI (0.90) compared to medical doctors (0.72) (18). Our model can increase the sensitivity to 0.95–0.99, which outperforms this ML-based ECG autodiagnostic system.

### 4.3. Advantages of Our Model

In this study, we reported three DL models for detecting STEMI based on 12-lead ECG. Deep learning-based classifiers presented high sensitivity and specificity in distinguishing STEMI cases with an AUC of 0.99. Convolutional neural network, LSTM, and CNN-LSTM performed similarly in this stage. A previous study established an ML-based method to detect myocardial relaxation abnormalities using 12-lead ECG (16). Miquel Alfaras used an ensemble of echo state networks (ESNs) as a classifier method (19).In the case of ESNs, reservoirs are current neural networks with random input and random connection weights between neurons. Thanks to the recurrence of the network, current reservoir responses depend on the previous state of the reservoir, yielding ESNs capable of performing context-dependent computations. The reservoir benefits from a high-dimensional nonlinear mapping of the input so that the reservoir response is easier to classify than the original input by means of a simple linear regression technique. This method is conducive to the diagnosis of arrhythmia, but there are still deficiencies in the diagnosis of myocardial infarction. Pranav Rajpurkar trained a 34-layer CNN to detect arrhythmia in arbitrary length ECG time series (20). They used residual connections and batch normalization to optimize the deep model. The depth increased both the nonlinearity of the computation and the size of the context window for each classification decision. The architecture of the model included 33 layers of convolution followed by a fully connected layer and a SoftMax layer. U Rajendra Acharya used an 11-layer deep CNN model for congestive heart failure diagnosis (21). The model consists of four convolutional, four max-pooling, and three fully connected layers. These layers make up the fundamental structure of CNN whereby convolution picks up distinctive features from the input ECG signal. The max-pooling operation reduces the dimensions of feature maps and simultaneously retains important and significant features of the input ECG signal. We used LSTM to extract contextual features without many CNN layers and fully connected layers. Jen Hong Tan used a stacked LSTM network with CNN to classify normal vs. coronary artery disease ECG signals (22). The algorithm first slices a 5 s ECG segment (with 1,285 data points) into 211 short segments. Each short segment consists of 24 data points. They perform two rounds of 1D convolution-max-pooling to extract the significant features in these segments. The resultant output is a set of 50 short segments, with each segment comprising 32 data points. This process reduces the number of data points for computation in LSTM (CNN for most of the time runs faster than LSTM). These segments are then fed to three layers of LSTM and a fully connected layer to perform the diagnosis. Our architecture is similar to their research, but we can handle a larger number of inputs (5,000 data points). Moreover, our LSTM layers are fewer and faster.

In contrast, our model used a 12-lead ECG, which can handle more comprehensive features. It is verified on the real-world ECG data set of myocardial infarctions, which is closer to reality. Our model uses LSTM+CNN, inputs original ECG data, and automatically extracts ECG features. It can use LSTM to extract the time dimension and CNN to extract spatial dimension information, with richer features, and the model has more classification capabilities.

### 4.4. Accurate Location of Culprit Vessels

We were the first to introduce DL models into culprit vessel recognition, which provided clues for localizing criminal vessels for CAG and PCI. Our CNN-LSTM model achieved an AUC of 0.95 in determining the location of LAD, which was slightly better than those of different levels of doctors. Among the different DL models, CNN-LSTM outperformed CNN and LSTM in locating LAD. Inferior myocardial infarction is more challenging to identify than anterior wall myocardial infarction by ECG. Although a few ECG diagnosis flow charts were highly accurate, they were challenging to remember, time-consuming, and not user-friendly for doctors at various levels (23). According to the CNN-LSTM architecture, the AUCs of the RCA and LCX were 0.81 and 0.63 in Tests 1 and 2, respectively. In other words, our models accurately located the culprit vessels using ECG.

### 4.5. Limitation

This study had some limitations. Firstly, this is a retrospective study. In order to verify the robust of the model, we applied an external dataset. Prospective ECG data will be used for AI research in the future. Secondly, 18-lead ECG should be performed routinely in patients with chest pain, but our AI model only uses 12-lead ECG data. Thirdly, patients with multi-vessel CAD were excluded because it was challenging to determine culprit more than two vessels based on ECG. One half of patients with STEMI have multivessel CAD. Compared with single-vessel CAD, the presence of multivessel CAD of STEMI is associated with poor prognosis, such as lower reperfusion success and higher adverse risk. Recently, a meta-analysis revealed multivessel PCI was associated with a low risk of reinfarction compared culprit vessel–only PCI in patients with STEMI (24). According to ECG, detecting STEMI and the location of vessel lesion (proximal or distal) and vessel size in patient with multivessel CAD required further analyses. Once patients with multivessel CAD are found through CAG, culprit, and non-culprit vessels will be intervened to minimize mortality and adverse events. For inferior and posterior myocardial infarction, the discrimination between LCX and RCA was inferior to doctors due to relatively small sample size of LCX (55 cases). Moreover, this AI-based ECG diagnosis algorithm should be validated in various ethnicities. Furthermore, the interpretation of DL algorithms required further investigation.

To our knowledge, this is the first DL approach to detect STEMI and culprit vessels based on real-world ECG data. The models are more sensitive and specific than doctors and auto ECG readers. Moreover, our DL algorithms are based on global cloud datasets and can provide a more accurate remote auxiliary diagnosis function, which will have great potential to be applied to clinical practice. In summary, this study demonstrates how to improve STEMI diagnosis by leveraging modern computing technology.

## Code Availability

The custom codes for the diagnosis and discrimination STEMI based on 12-lead ECG are available at the https://github.com/FarahHwang/AI_ECG_STEMI. The codes are available for download for non-commercial uses.

## Data Availability Statement

The original contributions presented in the study are included in the article/Supplementary Material, further inquiries can be directed to the corresponding author/s.

## Ethics Statement

The studies involving human participants were reviewed and approved by the Human Ethics Boards of The Third Affiliated Hospital of Sun Yat-sen University and Guangzhou First People's Hospital. Written informed consent for participation was not required for this study in accordance with the national legislation and the institutional requirements.

## Author Contributions

LW: conceptualization, methodology, original draft preparation, writing—review, editing, methodology, and funding acquisition. GH: conceptualization, methodology, software, and writing—review and editing. XY, MY, YLin, DL, BZ, YLiu, JZ, XH, RP, and SL: original draft preparation and methodology. LL and XL: conceptualization, methodology, and software. XQ and YC: conceptualization, methodology, writing—review and editing, funding acquisition, and supervision. LH: conceptualization, methodology, funding acquisition, and supervision. All authors contributed to the article and approved the submitted version.

## Funding

This study was funded by Guangdong Medical Research Foundation (A2019079), the National Natural Science Foundation of China (81770826 and 81370447), Science and Technology Planning Project of Guangdong Province (2016A050502014), National Key R&D Program (2018yfc1705105 and 2017YFA0105803), the 5010 Clinical Research Projects of Sun Yat-sen University (2015015), the Key Area R&D Program of Guangdong Province (2019B020227003), and the Science and Technology Plan Project of Guangzhou City (202007040003).

## Conflict of Interest

The authors declare that the research was conducted in the absence of any commercial or financial relationships that could be construed as a potential conflict of interest.

## Publisher's Note

All claims expressed in this article are solely those of the authors and do not necessarily represent those of their affiliated organizations, or those of the publisher, the editors and the reviewers. Any product that may be evaluated in this article, or claim that may be made by its manufacturer, is not guaranteed or endorsed by the publisher.
