# Supplementary material for: Deep Learning Networks Accurately Detect ST-Segment Elevation Myocardial Infarction and Culprit Vessel
Source: Front Cardiovasc Med. 2022 Mar 10;9:797207. doi: 10.3389/fcvm.2022.797207 (PMC8960131; doi:10.3389/fcvm.2022.797207)
Supplement: Supplementary file 1 [file Data_Sheet_1.PDF]

Supplementary Material

1 Supplementary Data

1. 1 CNN

A convolutional layer is similar to a single-layer MLP, except that it employs convolution rather than matrix multiplication (Extended data Fig. 1). A convolution operation for sequences is defined as:

$$y_t = \sum_{i=-K'}^{K'} w_{K'=1+i} * x_{t-i}$$

Where x_t and y_t are elements of input and output sequences at position t, respectively, and $w \in R^{KxC}$ is a kernel of size K=2K'+1.

The kernel is applied in this way at each position of the input sequence to produce the output sequence. For positions where kernel spans beyond the input sequence, we assume that the input is padded with zero vectors: $i_t = 0$ for $t \leq 0$ or $t > T$. The input and kernel elements themselves are vectors with multiple channels. The number of channels in ECG data is 12.

```
________________________________________________________________________________
Layer (type)                 Output Shape              Param #
================================================================================
conv1d_1 (Conv1D)            (None, 5000, 32)          800
________________________________________________________________________________
max_pooling1d_1 (MaxPooling1 (None, 2500, 32)          0
________________________________________________________________________________
conv1d_2 (Conv1D)            (None, 2500, 32)          2080
________________________________________________________________________________
max_pooling1d_2 (MaxPooling1 (None, 1250, 32)          0
________________________________________________________________________________
conv1d_3 (Conv1D)            (None, 1250, 48)          3120
________________________________________________________________________________
max_pooling1d_3 (MaxPooling1 (None, 625, 48)           0
________________________________________________________________________________
dropout_1 (Dropout)          (None, 625, 48)           0
________________________________________________________________________________
flatten_1 (Flatten)          (None, 30000)             0
________________________________________________________________________________
dense_1 (Dense)              (None, 2)                 60002
================================================================================
Total params: 66,002
Trainable params: 66,002
Non-trainable params: 0
```

1.1.1 LSTM

An LSTM cell holds the state s in two vectors: C(‘memoty’) and h (previous output). Input x and state vector h are concatenated before being processed in four steps (Extended data Fig. 2).

Input gate:

$$i_t = \sigma(w_i[h_{t-1}, x_t] + b_i)$$

(1)

Forget gate:

$$f_t = \sigma(w_f[h_{t-1}, x_t] + b_f)$$

(2)

Output gate:

$$o_t = \sigma(w_o[h_{t-1}, x_t] + b_o)$$

(3)

Candidate vector for cell state:

$$\acute{c}_t = \tanh(w_c[h_{t-1}, x_t] + b_c)$$

(4)

Where tanh is the element-wise hyperbolic tangent and σ is the logistic sigmoid function ($\sigma(t) = \frac{1}{1+e^{-t}}$). Matrices W and vectors b are parameters learned by the network.

The new cell state c_t and b_t, as well as the output y_t is given by:

Cell state:

$$c_t = f_t * c_{t-1} + i_t * \acute{c}_t \tag{5}$$

$$h_t = o_t * \tanh(c_t) \tag{6}$$

Where * is the element-wise vector multiplication (Extended data Fig. 3).

```
_________________________________________________________________
Layer (type)                 Output Shape              Param #
=================================================================
lstm_18 (LSTM)               (None, 5000, 100)         45200
_________________________________________________________________
dropout_33 (Dropout)         (None, 5000, 100)         0
_________________________________________________________________
lstm_19 (LSTM)               (None, 50)                30200
_________________________________________________________________
dropout_34 (Dropout)         (None, 50)                0
_________________________________________________________________
dense_31 (Dense)             (None, 3)                 153
=================================================================
Total params: 75,553
Trainable params: 75,553
Non-trainable params: 0
```

1.1.2 1. CNN-LSTM

1D CNNs are mainly utilized for audio and text recognition (as time series data). 1D-CNN are ideal tools for time-series recognition and prediction. The network has recently been established in state-of-the-art applications such as early diagnosis, structural health monitoring, as well as anomaly detection and identification. Considering that our data consisted of vibration signals (time-series type), we prefer to select 1D-CNN instead of traditional AI algorithms. In this network, the output of a convolutional layer (v_{ij}^{x}) at position x of the j_{th} feature map in the i_{th} layer is denoted as follows:

$$v_{ij}^{x} = g\left(b_{ij} + \sum_{m}\sum_{p=0}^{P_i-1} w_{im}^{p} v_{(i-1)m}^{x-p}\right) \quad (1)$$

Where m denotes the feature map in the previous layer ($(i-1)_{th}$ layer) connected to the current feature map; w_{im}^{p} denotes the indexed weight of position p in the m_{th} feature map; P_i is the width of the kernel toward the spectral dimension; b_{ij} is the bias of j_{th} feature map in the i_{th} layer. Meanwhile, g was the activation function. Typically, a pooling layer was employed after one or more CNN layers to ensure invariance by lowering the resolution of the feature maps. Each pooling layer

corresponds to the previous convolutional layer. The most common pooling operation was the max-pooling:

$$\bar{u}_n = \max_{1 \leq j \leq k} \left(u_n^j \right) \tag{2}$$

Where u_n^j is the j_{th} element of the n_{th} patch, $\bar{u}_n$ is the sample of the n_{th} patch built by max-pooling, and “k” was the size of the n_{th} patch.

An LSTM cell held the state s in two vectors: C(‘memoty’) and h (previous output). Input x and state vector h were concatenated before being processed in four steps:

Input gate:

$$i_t = \sigma(w_i[h_{t-1}, x_t] + b_i)$$

(1)

Forget gate:

$$f_t = \sigma(w_f[h_{t-1}, x_t] + b_f)$$

(2)

Output gate:

$$o_t = \sigma(w_o[h_{t-1}, x_t] + b_o)$$

(3)

Candidate vector for cell state:

$$\acute{c}_t = \tanh(w_c[h_{t-1}, x_t] + b_c)$$

(4)

Where tanh is the element-wise hyperbolic tangent and σ is the logistic sigmoid function ($\sigma(t) = \frac{1}{1+e^{-t}}$). Matrices W and vectors b are parameters learned by the network.

The new cell state c_t and b_t, as well as the output y_t is given by:

Cell state:

$$c_t = f_t * c_{t-1} + i_t * \acute{c}_t$$

(5)

$$h_t = o_t * \tanh(c_t)$$

(6)

Where * is the element-wise vector multiplication.

```
_________________________________________________________________
Layer (type)                 Output Shape              Param #
=================================================================
time_distributed_1 (TimeDist (None, 100, 50, 16)       400
_________________________________________________________________
time_distributed_2 (TimeDist (None, 100, 25, 16)       0
_________________________________________________________________
time_distributed_3 (TimeDist (None, 100, 25, 16)       528
_________________________________________________________________
time_distributed_4 (TimeDist (None, 100, 12, 16)       0
_________________________________________________________________
time_distributed_5 (TimeDist (None, 100, 12, 48)       1584
_________________________________________________________________
time_distributed_6 (TimeDist (None, 100, 6, 48)        0
_________________________________________________________________
time_distributed_7 (TimeDist (None, 100, 6, 48)        0
_________________________________________________________________
time_distributed_8 (TimeDist (None, 100, 288)          0
_________________________________________________________________
lstm_1 (LSTM)                (None, 100)               155600
_________________________________________________________________
dense_1 (Dense)              (None, 2)                 202
=================================================================
Total params: 158,314
Trainable params: 158,314
Non-trainable params: 0
```

Layer (type)	Output Shape	Param #
time_distributed_81 (TimeDis	(None, 50, 100, 16)	400
time_distributed_82 (TimeDis	(None, 50, 50, 16)	0
time_distributed_83 (TimeDis	(None, 50, 50, 16)	528
time_distributed_84 (TimeDis	(None, 50, 25, 16)	0
time_distributed_85 (TimeDis	(None, 50, 25, 32)	1056
time_distributed_86 (TimeDis	(None, 50, 12, 32)	0
time_distributed_87 (TimeDis	(None, 50, 12, 32)	0
time_distributed_88 (TimeDis	(None, 50, 384)	0
lstm_11 (LSTM)	(None, 50)	87000
dense_16 (Dense)	(None, 3)	153

Total params: 89,137
Trainable params: 89,137
Non-trainable params: 0

1.1.3 Extended data Figure

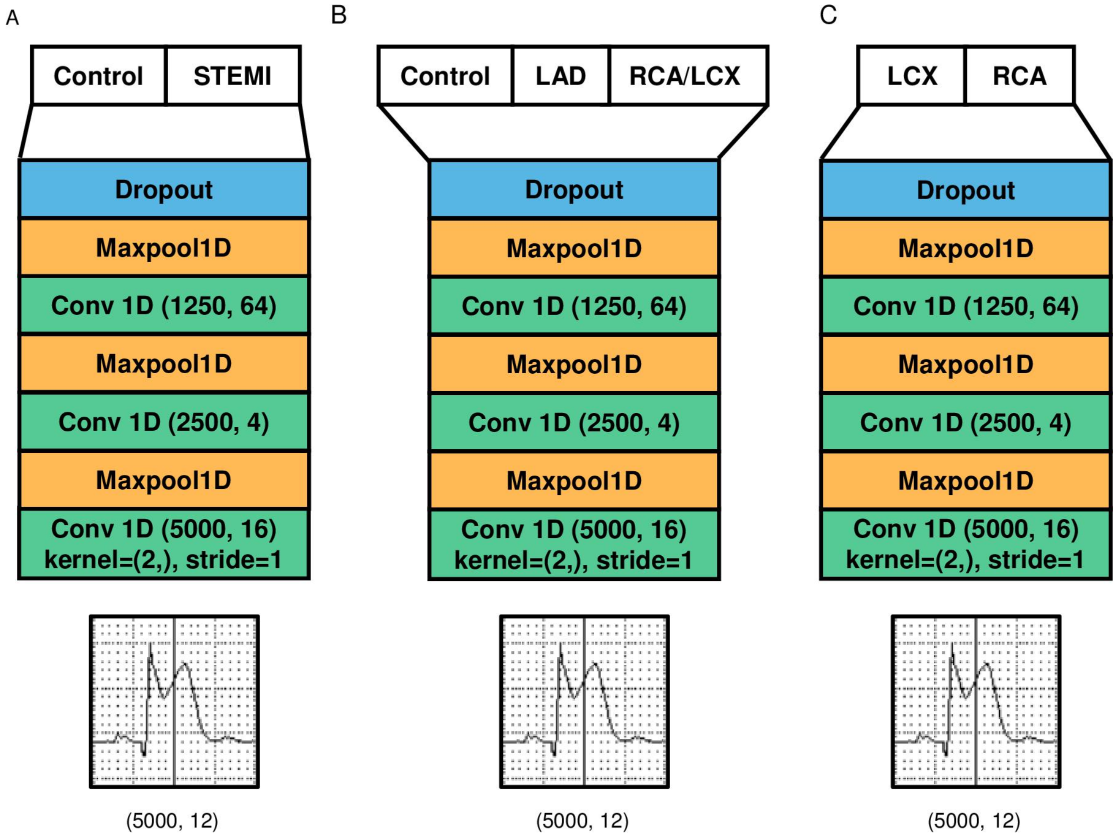

Supplementary Fig. 1 Schematic of CNN architecture.(A) Diagnostic performance of CNN to predict Control and STEMI. (B) Diagnostic performance of CNN to predict Control,LAD and RCA/LCX.(C)Diagnostic performance of CNN to predict RCA and LCX.

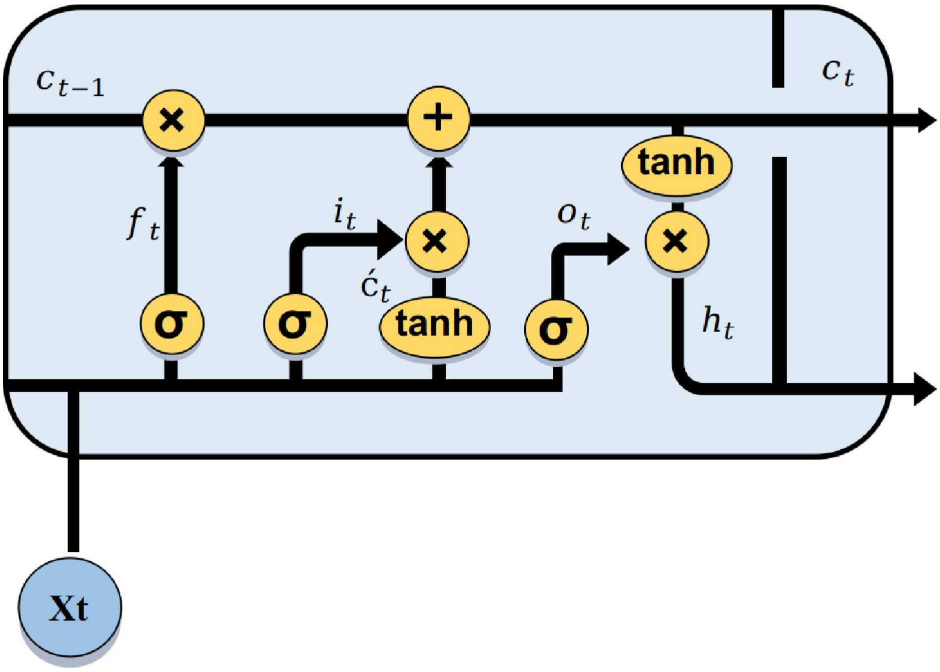

Supplementary Fig. 2 Schematic of LSTM architecture.

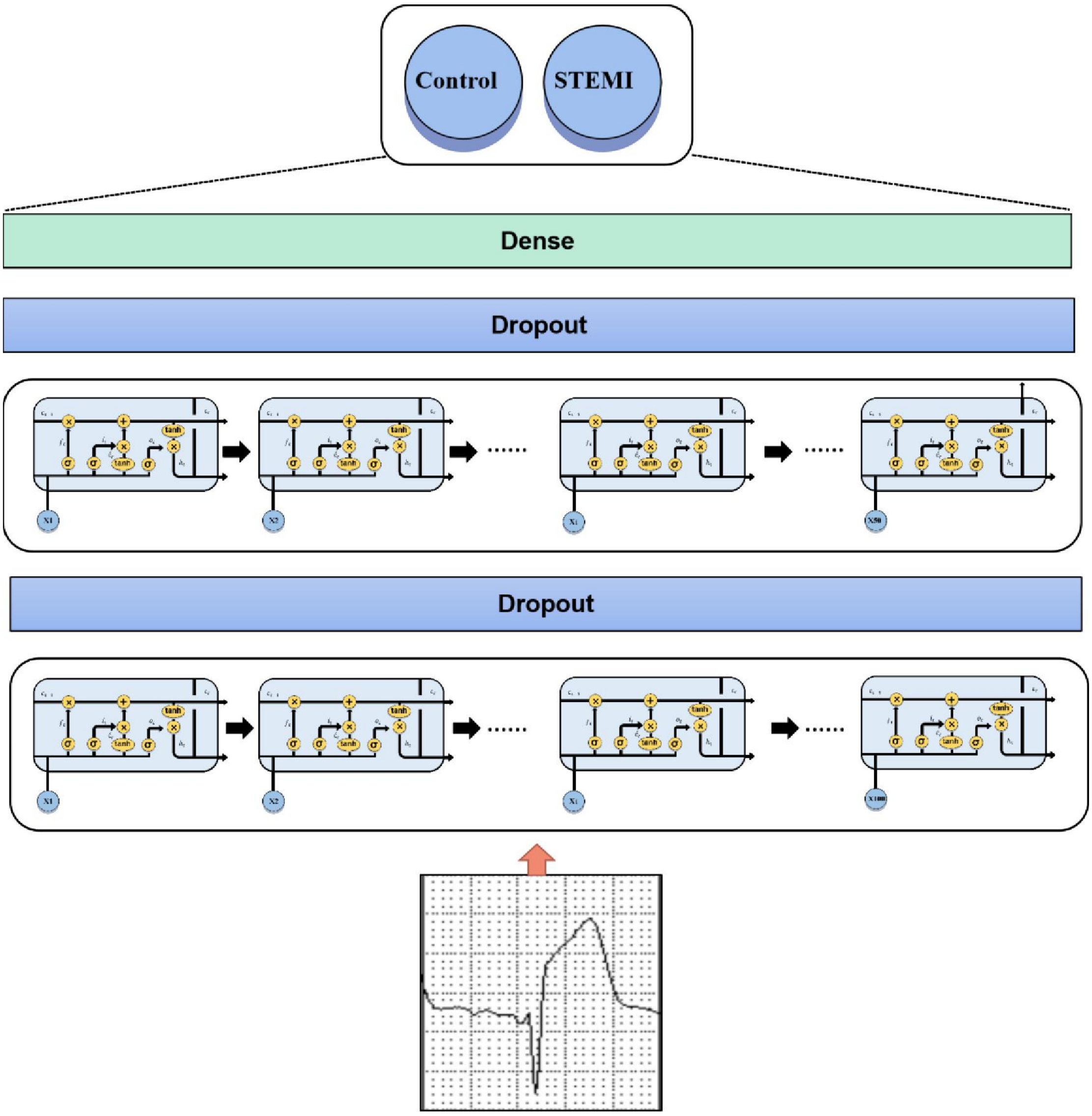

Supplementary Fig. 3 Flowchart of CNN-LSTM architecture.

2 Supplementary Figures and Tables

Supplementary Table 1 Abbreviation

Abbreviation	Interpretation
CVD family history	cardiovascular disease family history
WBC	white blood cell count

RBC	red blood cell count
HGB	hemoglobin concentration
PLT	platelet count
ALB	albumin
GLB	globulin
K	potassium
Na	sodium
Ca	calcium
GLU	fasting blood glucose
BUN	blood urea nitrogen
CREAT	Serum creatinine
CHOL	total cholesterol
TG	triglyceride
HDL-C	high density lipoprotein cholesterol
LDL-C	low density lipoprotein cholesterol
CK-MB	creatine kinase-MB
Minimum HR	minimum heart rate
Maximun HR	maximum heart rate
Average HR	average heart rate
PR	PR interval
P wave	P wave duration
QRS complex	QRS complex
T wave	T wave duration
QT interval	QT interval
QTc interval	QTc interval

QRS axis	QRS axis
RV1+SV5	R wave amplitude of V1 plus S wave amplitude of V5
RV5+SV1	R wave amplitude of V5 plus S wave amplitude of V1
R-PB	Peak R wave to the begining of P wave
R-P	Peak R wave to peak P wave
R-PE	Peak R wave to the ending of P wave
R-Q	Peak R wave to peak Q wave
R-S	Peak R wave to peak S wave
R-TB	Peak R wave to the begining of T wave
R-T	Peak R wave to the peak of T wave
R-TE	Peak R wave to the ending of T wave
Q-P	Peak Q wave to peak P wave
S-T	Peak S wave to peak P wave
Q-PB	Peak Q wave to the begining of P wave
S-TE	Peak S wave to the ending of T wave
I (PB)	the amplitude of the begining of P wave in Lead I
II(PB)	the amplitude of the begining of P wave in Lead II
III(PB)	the amplitude of the begining of P wave in Lead III
aVR(PB)	the amplitude of the begining of P wave in Lead aVR
aVL(PB)	the amplitude of the begining of P wave in Lead aVL
aVF(PB)	the amplitude of the begining of P wave in Lead aVF
V1(PB)	the amplitude of the begining of P wave in Lead V1
V2(PB)	the amplitude of the begining of P wave in Lead V2
V3(PB)	the amplitude of the begining of P wave in Lead V3
V4(PB)	the amplitude of the begining of P wave in Lead V4

V5(PB)	the amplitude of the begining of P wave in Lead V5
V6(PB)	the amplitude of the begining of P wave in Lead V6
I (PE)	the amplitude of the ending of P wave in Lead I
II(PE)	the amplitude of the ending of P wave in Lead II
III(PE)	the amplitude of the ending of P wave in Lead III
aVR(PE)	the amplitude of the ending of P wave in Lead aVR
aVL(PE)	the amplitude of the ending of P wave in Lead aVL
aVF(PE)	the amplitude of the ending of P wave in Lead aVF
V1(PE)	the amplitude of the ending of P wave in Lead V1
V2(PE)	the amplitude of the ending of P wave in Lead V2
V3(PE)	the amplitude of the ending of P wave in Lead V3
V4(PE)	the amplitude of the ending of P wave in Lead V4
V5(PE)	the amplitude of the ending of P wave in Lead V5
V6(PE)	the amplitude of the ending of P wave in Lead V6
I (Q)	the Q wave amplitude in Lead I
II(Q)	the Q wave amplitude in Lead II
III(Q)	the Q wave amplitude in Lead III
aVR(Q)	the Q wave amplitude in Lead aVR
aVL(Q)	the Q wave amplitude in Lead aVL
aVF(Q)	the Q wave amplitude in Lead aVF
V1(Q)	the Q wave amplitude in Lead V1
V2(Q)	the Q wave amplitude in Lead V2
V3(Q)	the Q wave amplitude in Lead V3
V4(Q)	the Q wave amplitude in Lead V4
V5(Q)	the Q wave amplitude in Lead V5

V6(Q)	the Q wave amplitude in Lead V6
I (R)	the R wave amplitude in Lead I
II(R)	the Rwave amplitude in Lead II
III(R)	the R wave amplitude in Lead III
aVR(R)	the R wave amplitude in Lead aVR
aVL(R)	the R wave amplitude in Lead aVL
aVF(R)	the R wave amplitude in Lead aVF
V1(R)	the R wave amplitude in Lead V1
V2(R)	the R wave amplitude in Lead V2
V3(R)	the R wave amplitude in Lead V3
V4(R)	the R wave amplitude in Lead V4
V5(R)	the R wave amplitude in Lead V5
V6(R)	the R wave amplitude in Lead V6
I (S)	the S wave amplitude in Lead I
II(S)	the S wave amplitude in Lead II
III(S)	the S wave amplitude in Lead III
aVR(S)	the S wave amplitude in Lead aVR
aVL(S)	the S wave amplitude in Lead aVL
aVF(S)	the S wave amplitude in Lead aVF
V1(S)	the S wave amplitude in Lead V1
V2(S)	the S wave amplitude in Lead V2
V3(S)	the S wave amplitude in Lead V3
V4(S)	the S wave amplitude in Lead V4
V5(S)	the S wave amplitude in Lead V5
V6(S)	the S wave amplitude in Lead V6

I (R')	the R' wave amplitude in Lead I
II(R')	the R' wave amplitude in Lead II
III(R')	the R' wave amplitude in Lead III
aVR(R')	the R' wave amplitude in Lead aVR
aVL(R')	the R' wave amplitude in Lead aVL
aVF(R')	the R' wave amplitude in Lead aVF
V1(R')	the R' wave amplitude in Lead V1
V2(R')	the R' wave amplitude in Lead V2
V3(R')	the R' wave amplitude in Lead V3
V4(R')	the R' wave amplitude in Lead V4
V5(R')	the R' wave amplitude in Lead V5
V6(R')	the R' wave amplitude in Lead V6
I (S')	the S' wave amplitude in Lead I
II(S')	the S' wave amplitude in Lead II
III(S')	the S' wave amplitude in Lead III
aVR(S')	the S' wave amplitude in Lead aVR
aVL(S')	the S' wave amplitude in Lead aVL
aVF(S')	the S' wave amplitude in Lead aVF
V1(S')	the S' wave amplitude in Lead V1
V2(S')	the S' wave amplitude in Lead V2
V3(S')	the S' wave amplitude in Lead V3
V4(S')	the S' wave amplitude in Lead V4
V5(S')	the S' wave amplitude in Lead V5
V6(S')	the S' wave amplitude in Lead V6
I (TB)	the S' wave amplitude in Lead I

II(TB)	the S' wave amplitude in Lead II
III(TB)	the S' wave amplitude in Lead III
aVR(TB)	the S' wave amplitude in Lead aVR
aVL(TB)	the S' wave amplitude in Lead aVL
aVF(TB)	the S' wave amplitude in Lead aVF
V1(TB)	the S' wave amplitude in Lead V1
V2(TB)	the S' wave amplitude in Lead V2
V3(TB)	the S' wave amplitude in Lead V3
V4(TB)	the S' wave amplitude in Lead V4
V5(TB)	the S' wave amplitude in Lead V5
V6(TB)	the S' wave amplitude in Lead V6
I (TE)	the amplitude of the begining of T wave in Lead I
II(TE)	the amplitude of the begining of T wave in Lead II
III(TE)	the amplitude of the begining of T wave in Lead III
aVR(TE)	the amplitude of the begining of T wave in Lead aVR
aVL(TE)	the amplitude of the begining of T wave in Lead aVL
aVF(TE)	the amplitude of the begining of T wave in Lead aVF
V1(TE)	the amplitude of the begining of T wave in Lead V1
V2(TE)	the amplitude of the begining of T wave in Lead V2
V3(TE)	the amplitude of the begining of T wave in Lead V3
V4(TE)	the amplitude of the begining of T wave in Lead V4
V5(TE)	the amplitude of the begining of T wave in Lead V5
V6(TE)	the amplitude of the begining of T wave in Lead V6
I (ST20)	the amplitude of ST segment at 20 ms from J point in Lead I
II(ST20)	the amplitude of ST segment at 20 ms from J point in Lead II

III(ST20)	the amplitude of ST segment at 20 ms from J point in Lead III
aVR(ST20)	the amplitude of ST segment at 20 ms from J point in Lead aVR
aVL(ST20)	the amplitude of ST segment at 20 ms from J point in Lead aVL
aVF(ST20)	the amplitude of ST segment at 20 ms from J point in Lead aVF
V1(ST20)	the amplitude of ST segment at 20 ms from J point in Lead V1
V2(ST20)	the amplitude of ST segment at 20 ms from J point in Lead V2
V3(ST20)	the amplitude of ST segment at 20 ms from J point in Lead V3
V4(ST20)	the amplitude of ST segment at 20 ms from J point in Lead V4
V5(ST20)	the amplitude of ST segment at 20 ms from J point in Lead V5
V6(ST20)	the amplitude of ST segment at 20 ms from J point in Lead V6
I (ST40)	the amplitude of ST segment at 40 ms from J point in Lead I
II(ST40)	the amplitude of ST segment at 40 ms from J point in Lead II
III(ST40)	the amplitude of ST segment at 40 ms from J point in Lead III
aVR(ST40)	the amplitude of ST segment at 40 ms from J point in Lead aVR
aVL(ST40)	the amplitude of ST segment at 40 ms from J point in Lead aVL
aVF(ST40)	the amplitude of ST segment at 40 ms from J point in Lead aVF
V1(ST40)	the amplitude of ST segment at 40 ms from J point in Lead V1
V2(ST40)	the amplitude of ST segment at 40 ms from J point in Lead V2
V3(ST40)	the amplitude of ST segment at 40 ms from J point in Lead V3
V4(ST40)	the amplitude of ST segment at 40 ms from J point in Lead V4
V5(ST40)	the amplitude of ST segment at 40 ms from J point in Lead V5
V6(ST40)	the amplitude of ST segment at 40 ms from J point in Lead V6
I (ST60)	the amplitude of ST segment at 60 ms from J point in Lead I
II(ST60)	the amplitude of ST segment at 60 ms from J point in Lead II
III(ST60)	the amplitude of ST segment at 60 ms from J point in Lead III

aVR(ST60)	the amplitude of ST segment at 60 ms from J point in Lead aVR
aVL(ST60)	the amplitude of ST segment at 60 ms from J point in Lead aVL
aVF(ST60)	the amplitude of ST segment at 60 ms from J point in Lead aVF
V1(ST60)	the amplitude of ST segment at 60 ms from J point in Lead V1
V2(ST60)	the amplitude of ST segment at 60 ms from J point in Lead V2
V3(ST60)	the amplitude of ST segment at 60 ms from J point in Lead V3
V4(ST60)	the amplitude of ST segment at 60 ms from J point in Lead V4
V5(ST60)	the amplitude of ST segment at 60 ms from J point in Lead V5
V6(ST60)	the amplitude of ST segment at 60 ms from J point in Lead V6
I (ST80)	the amplitude of ST segment at 80 ms from J point in Lead I
II(ST80)	the amplitude of ST segment at 80 ms from J point in Lead II
III(ST80)	the amplitude of ST segment at 80 ms from J point in Lead III
aVR(ST80)	the amplitude of ST segment at 80 ms from J point in Lead aVR
aVL(ST80)	the amplitude of ST segment at 80 ms from J point in Lead aVL
aVF(ST80)	the amplitude of ST segment at 80 ms from J point in Lead aVF
V1(ST80)	the amplitude of ST segment at 80 ms from J point in Lead V1
V2(ST80)	the amplitude of ST segment at 80 ms from J point in Lead V2
V3(ST80)	the amplitude of ST segment at 80 ms from J point in Lead V3
V4(ST80)	the amplitude of ST segment at 80 ms from J point in Lead V4
V5(ST80)	the amplitude of ST segment at 80 ms from J point in Lead V5
V6(ST80)	the amplitude of ST segment at 80 ms from J point in Lead V6

Supplementary Table 2 Baseline characteristics between Cohort 1 and Cohort 2

	Cohort 1	Cohort 2	*P* value
n	793	90	
Age, year, mean(SD)	57.3±13.3	59.2±10.6	0.150
Gender(female)	271(354.2%)	25(27.8%)	0.241
Diabetes mellitus	163(20.6%)	9(10.0%)	0.016
Hypertension	344(43.4%)	17(18.9%)	0.000
Chronic kidney disease	17(2.1%)	4(4.4%)	0.259
CVD family history	39(4.9%)	5(5.6%)	0.797
WBC(*$10^{9/L}$)	8.14±3.21	7.24±2.46	0.002
RBC(*10^{9}/L)	4.33±0.86	4.33±0.79	0.933
HGB(g/ml)	129.62±17.62	127.2±21.78	0.311
PLT(*10^{9}/L)	210.30±59.52	226.76±65.87	0.014
ALB(g/L)	38.94±4.51	39.51±4.85	0.259
GLB(g/L)	24.69±4.25	26.51±4.68	0.000
K(mmol/L)	3.61±0.41	4.19±0.65	0.000
Na(mmol/L)	137.20±4.69	141.72±3.31	0.000
Ca(mmol/L)	2.15±0.83	2.07±0.25	0.362
GLU(mmol/L)	6.92±3.03	6.78±1.89	0.530
BUN(mmol/L)	5.21±2.67	7.07±3.88	0.000
CREAT(mmol/L)	78.42±54.09	91.27±42.09	0.030
CHOL(mmol/L)	4.54±1.2	4.22±1.34	0.018

TG(mmol/L)	1.64±1.09	1.40±1.13	0.050
HDL(mmol/L)	1.05±0.31	1.08±0.34	0.466
LDL(mmol/L)	2.88±1.01	2.63±1.12	0.030
CK-MB(U/L)	18.6±42.57	11.66±12.79	0.010

* vs Cohort 1 $P<0.05$, ** vs Cohort 1 $P<0.01$

Supplementary Table 3 Baseline characteristics among different locations of culprit vessels

	Control	LAD	RCA	LCX	*P* value
n	506	194	128	55	
Age(years)	55.33±12.49	60.89±13.04	60.49±13.03	58.64±14.18	0.000
Gender(female)	227(44.9%)	38(19.6%)	22(17.2%)	9(16.4%)	0.000
Diabetes mellitus	101(20%)	31(16.0%)	30(23.4%)	10(18.2%)	0.133
Hypertension	226(44.7%)	65(33.5%)	53(41.4%)	17(30.9%)	0.022
Chronic kidney disease	12(2.4%)	6(3.1%)	1(0.8%)	2(3.6%)	0.530
CVD family history	23(4.5%)	11(5.7%)	7(5.5%)	3(5.5%)	0.922
White blood cell (*10^9/L)	7.33±2.98	8.9±3.14	9.39±3.16	8.48±2.87	0.000
Red blood cell(*10^9/L)	4.28±0.95	4.39±0.69	4.40±0.70	4.38±0.66	0.271
Hemoglobin(g/ml)	128.05±16.84	132.07±20.39	129.41±19.42	132±16.43	0.042
Platelet (*10^9/L)	207.21±59.3	219.29±60.74	213.95±61.76	225.42±62.19	0.031
ALB(g/L)	39.29±4.43	38.20±4.96	39.02±4.42	39.11±4.22	0.043
Globulin(g/L)	24.65±3.91	25.19±4.66	25.27±5.34	24.96±4.19	0.322
Potassium(mmol/L)	3.58±0.42	3.79±0.56	3.75±0.5	3.84±0.43	0.000
Sodium(mmol/L)	137.47±4.61	137.51±5.17	138.3±4.06	138.37±5.98	0.208
Ca(mmol/L)	2.15±0.31	2.07±0.36	2.01±0.47	2.58±2.84	0.000
Fasting glucose(mmol/L)	6.71±2.99	7.28±2.83	7.12±2.86	6.91±2.76	0.107
Blood urea nitrogen(mmol/L)	5.12±2.74	5.85±3.39	5.57±2.35	5.94±2.89	0.007
Serum creatinine(mmol/L)	81.71±58.42	76.11±45.47	73.23±33.61	89.39±61.97	0.148
CHOL(mmol/L)	4.52±1.23	4.62±1.19	4.32±1.19	4.50±1.35	0.192

TG(mmol/L)	1.59±1.08	1.60±1.07	1.69±1.21	1.71±1.07	0.745
HDL(mmol/L)	1.09±0.32	1.03±0.33	0.97±0.26	0.97±0.3	0.000
LDL(mmol/L)	2.79±1.01	3.02±1.05	2.84±0.99	2.85±1.11	0.073
CK-MB(U/L)	6.76±6.31	37.31±64.95	27.77±49.31	28.78±53.63	0.000

CVD cardiovascular disease,*CHOL* total cholesterol, *HDL-C* high density lipoprotein cholesterol, *LDL-C* low density lipoprotein cholesterol, *CK-MB* serum creatine kinase MB, *LAD* left anterior descending artery, *RCA* right coronary artery, *LCX* left circumflex artery.

P value vs control

Supplementary Table 4 ECG features between patients with or without STEMI

	Control	STEMI	*P* value
n	506	377	
Minimum HR(bpm)	69.45±12.65	71.81±17.39	0.026
Maximun HR(bpm)	78.72±20.03	87.59±26.61	0.000
Average HR(bpm)	73.24±12.57	78.41±17.46	0.000
PR(ms)	151.72±24.47	155.28±27.05	0.041
P wave(ms)	108.66±22.01	104.79±21.99	0.010
QRS complex(ms)	102.87±11.02	105.83±14.2	0.001
T wave(ms)	183.65±21.67	175.66±34.69	0.000
QT interval(ms)	386.41±32.36	393.85±43.63	0.005
QTc interval(ms)	424.23±26.77	445.14±35.62	0.000
QRS axis(degree)	43.16±37.62	31.41±66.12	0.002
RV1+SV5 (mv)	0.54±0.54	0.41±0.54	0.000
RV5+SV1 (mv)	2.29±0.74	1.76±0.96	0.000
R-PB(ms)	201.11±25.66	204.90±27.66	0.038
R-P(ms)	137.56±21.22	148.89±29.25	0.000
R-PE(ms)	92.29±12.82	99.4±21.19	0.000
R-Q(ms)	49.39±3.97	49.69±5.40	0.333
R-S(ms)	53.48±8.26	56.13±11.53	0.000
R-TB(ms)	153.37±23.19	168.49±31.72	0.000
R-T(ms)	264.55±39.61	266.85±39.79	0.394

R-TE(ms)	337.02±31.99	344.15±44.03	0.008
Q-P(ms)	88.43±20.2	100.30±28.65	0.000
S-T(ms)	211.07±39.72	210.72±41.51	0.899
Q-PB(ms)	151.70±24.45	155.09±26.62	0.050
S-TE(ms)	283.54±32.67	288.02±46.17	0.109
I (PB)((mv))	0.0579±0.0263	0.06230±0.0300	0.025
II(PB)(mv)	0.1054±0.0433	0.0987±0.0467	0.029
III(PB)(mv)	0.0695±0.0424	0.0611±0.0387	0.002
aVR(PB)(mv)	0.0001±0.0013	0.0007±0.0058	0.047
aVL(PB)(mv)	0.0241±0.0238	0.0286±0.0268	0.009
aVF(PB)(mv)	0.0833±0.0427	0.0761±0.0412	0.012
V1(PB)(mv)	0.0397±0.0265	0.033±0.0294	0.001
V2(PB)(mv)	0.0561±0.0281	0.0451±0.038	0.000
V3(PB)(mv)	0.0639±0.0258	0.0538±0.0317	0.000
V4(PB)(mv)	0.0638±0.0239	0.0546±0.0302	0.000
V5(PB)(mv)	0.0607±0.0228	0.0537±0.0288	0.000
V6(PB)(mv)	0.0565±0.0222	0.0528±0.0287	0.036
I (PE)(mv)	-0.0001±0.0016	-0.0011±0.0083	0.017
II(PE)(mv)	-0.0004±0.0038	-0.001±0.0081	0.207
III(PE)(mv)	-0.0096±0.0189	-0.0127±0.0229	0.038
aVR(PE)(mv)	-0.0757±0.027	-0.0741±0.0329	0.447
aVL(PE)(mv)	-0.0002±0.0230	-0.0161±0.0209	0.008
aVF(PE)(mv)	-0.0012±0.0067	-0.0020±0.0104	0.171
V1(PE)(mv)	-0.0294±0.029	-0.0418±0.0331	0.000

V2(PE)(mv)	-0.0065±0.0166	-0.0119±0.0223	0.000
V3(PE)(mv)	-0.0019±0.0086	-0.0061±0.0181	0.000
V4(PE)(mv)	-0.0009±0.0059	-0.0037±0.0130	0.000
V5(PE)(mv)	-0.0008±0.0051	-0.0025±0.0111	0.005
V6(PE)(mv)	-0.0005±0.0038	-0.0008±0.0065	0.351
I (Q)(mv)	-0.0264±0.0320	-0.0297±0.0881	0.484
II(Q)(mv)	-0.0281±0.0398	-0.0829±0.1122	0.000
III(Q)(mv)	-0.0562±0.1088	-0.2469±0.3132	0.000
aVR(Q)(mv)	-0.3847±0.3408	-0.2545±0.2822	0.000
aVL(Q)(mv)	-0.0431±0.0698	-0.0408±0.1021	0.691
aVF(Q)(mv)	-0.0335±0.0602	-0.1378±0.1810	0.000
V1(Q)(mv)	-0.0463±0.2050	-0.3494±0.5002	0.000
V2(Q)(mv)	-0.0142±0.1415	-0.5121±0.8393	0.000
V3(Q)(mv)	-0.0134±0.1467	-0.3851±0.6875	0.000
V4(Q)(mv)	-0.0125±0.0629	-0.1499±0.3503	0.000
V5(Q)(mv)	-0.0234±0.0418	-0.0769±0.1636	0.000
V6(Q)(mv)	-0.0302±0.0364	-0.0450±0.0730	0.000
I (R)(mv)	0.6059±0.2791	0.5359±0.3180	0.001
II(R)(mv)	0.7712±0.3213	0.4364±0.3038	0.000
III(R)(mv)	0.3672±0.3255	0.2637±0.2722	0.000
aVR(R)(mv)	0.0728±0.0847	0.0808±0.0992	0.207
aVL(R)(mv)	0.3281±0.2544	0.4137±0.3063	0.000
aVF(R)(mv)	0.5190±0.3331	0.2982±0.2769	0.000
V1(R)(mv)	0.2283±0.1865	0.1555±0.2425	0.000

V2(R)(mv)	0.6522±0.3491	0.4596±0.5676	0.000
V3(R)(mv)	1.0015±0.5048	0.6888±0.7877	0.000
V4(R)(mv)	1.5120±0.6068	0.947±0.9074	0.000
V5(R)(mv)	1.4901±0.5612	0.968±0.7461	0.000
V6(R)(mv)	1.2020±0.4529	0.8065±0.5610	0.000
I (S)(mv)	-0.1014±0.1142	-0.1043±0.1277	0.734
II(S)(mv)	-0.0929±0.1345	-0.0946±0.1384	0.855
III(S)(mv)	-0.1738±0.2358	-0.1302±0.2429	0.007
aVR(S)(mv)	-0.2839±0.3875	-0.1966±0.2726	0.000
aVL(S)(mv)	-0.1518±0.1614	-0.1277±0.1609	0.028
aVF(S)(mv)	-0.0979±0.1453	-0.0844±0.1525	0.181
V1(S)(mv)	-0.8266±0.3991	-0.7922±0.4827	0.261
V2(S)(mv)	-1.2228±0.6015	-0.8411±0.8050	0.000
V3(S)(mv)	-0.9097±0.6044	-0.6709±0.6364	0.000
V4(S)(mv)	-0.6115±0.4739	-0.5253±0.4562	0.007
V5(S)(mv)	-0.3514±0.3037	-0.3295±0.2860	0.272
V6(S)(mv)	-0.1760±0.1940	-0.1447±0.1794	0.015
I (R')(mv)	0.0001±0.0022	0.0009±0.0161	0.321
II(R')(mv)	0.0019±0.013	0.0053±0.0322	0.056
III(R')(mv)	0.0485±0.1103	0.0197±0.0671	0.000
aVR(R')(mv)	0.0174±0.0507	0.0323±0.072	0.001
aVL(R')(mv)	0.0061±0.0225	0.0031±0.0191	0.028
aVF(R')(mv)	0.0150±0.0588	0.0131±0.0527	0.604
V1(R')(mv)	0.0188±0.1287	0.0076±0.0618	0.087

V2(R')(mv)	0.0016±0.0179	0.003±0.0237	0.324
V3(R')(mv)	0.0028±0.0325	0.0041±0.0481	0.641
V4(R')(mv)	0.0017±0.0146	0.0072±0.0704	0.137
V5(R')(mv)	0.0012±0.0139	0.0007±0.0125	0.640
V6(R')(mv)	0.0002±0.0028	0.0004±0.0069	0.541
I (S')(mv)	-0.0001±0.0018	0.0000±0.0000	0.388
II(S')(mv)	0.0000±0.0000	-0.0011±0.015	0.159
III(S')(mv)	-0.0062±0.0349	-0.0034±0.0294	0.197
aVR(S')(mv)	-0.0001±0.0018	0.0000±0.0000	0.388
aVL(S')(mv)	-0.0005±0.0096	-0.0001±0.0016	0.384
aVF(S')(mv)	-0.0024±0.0246	-0.0051±0.0402	0.243
V1(S')(mv)	-0.0022±0.0238	0.0000±0.0000	0.037
V2(S')(mv)	-0.0013±0.0194	-0.0075±0.1034	0.255
V3(S')(mv)	-0.0002±0.004	-0.0027±0.044	0.277
V4(S')(mv)	-0.0003±0.0058	-0.0067±0.0673	0.063
V5(S')(mv)	0.0000±0.0000	-0.0006±0.0124	0.318
V6(S')(mv)	0.0000±0.0000	-0.0003±0.0052	0.318
I (TB)(mv)	0.1331±0.0704	0.0810±0.0801	0.000
II(TB)(mv)	0.1600±0.0846	0.0874±0.0897	0.000
III(TB)(mv)	0.0566±0.063	0.0699±0.0903	0.014
aVR(TB)(mv)	0.0011±0.0089	0.0116±0.0311	0.000
aVL(TB)(mv)	0.0607±0.0586	0.0727±0.0901	0.025
aVF(TB)(mv)	0.0999±0.0729	0.0742±0.0857	0.000
V1(TB)(mv)	0.0596±0.0916	0.0824±0.0937	0.000

V2(TB)(mv)	0.3247±0.2075	0.2887±0.2604	0.027
V3(TB)(mv)	0.3171±0.2112	0.2232±0.2456	0.000
V4(TB)(mv)	0.3108±0.1970	0.1483±0.1876	0.000
V5(TB)(mv)	0.2583±0.1540	0.0944±0.1172	0.000
V6(TB)(mv)	0.2045±0.1155	0.0766±0.0976	0.000
I (TE)(mv)	0.1331±0.0704	0.081±0.0801	0.000
II(TE)(mv)	0.1600±0.0846	0.0874±0.0897	0.000
III(TE)(mv)	0.0566±0.063	0.0699±0.0903	0.014
aVR(TE)(mv)	0.0011±0.0089	0.0116±0.0311	0.000
aVL(TE)(mv)	0.0607±0.0586	0.0727±0.0901	0.025
aVF(TE)(mv)	0.0999±0.0729	0.0742±0.0857	0.000
V1(TE)(mv)	0.0596±0.0916	0.0824±0.0937	0.000
V2(TE)(mv)	0.3247±0.2075	0.2887±0.2604	0.027
V3(TE)(mv)	0.3171±0.2112	0.2232±0.2456	0.000
V4(TE)(mv)	0.3108±0.1970	0.1483±0.1876	0.000
V5(TE)(mv)	0.2583±0.1540	0.0944±0.1172	0.000
V6(TE)(mv)	0.2045±0.1155	0.0766±0.0976	0.000
I (ST20)(mv)	0.0018±0.0199	-0.0118±0.0457	0.000
II(ST20)(mv)	0.0057±0.0295	0.0238±0.0769	0.000
III(ST20)(mv)	0.0025±0.0226	0.0353±0.1005	0.000
aVR(ST20)(mv)	-0.0036±0.0222	-0.0054±0.0378	0.399
aVL(ST20)(mv)	-0.0005±0.0147	-0.0227±0.0674	0.000
aVF(ST20)(mv)	0.0038±0.0243	0.0284±0.0859	0.000
V1(ST20)(mv)	0.0413±0.0374	0.0538±0.0878	0.009

V2(ST20)(mv)	0.0854±0.0657	0.1056±0.1509	0.015
V3(ST20)(mv)	0.0555±0.0622	0.0788±0.1576	0.007
V4(ST20)(mv)	0.0235±0.0490	0.0351±0.1257	0.090
V5(ST20)(mv)	0.0037±0.0389	0.0058±0.0839	0.646
V6(ST20)(mv)	-0.0013±0.0319	-0.0008±0.0562	0.880
I (ST40)(mv)	0.0092±0.0217	-0.0059±0.0475	0.000
II(ST40)(mv)	0.0107±0.031	0.029±0.0769	0.000
III(ST40)(mv)	0.0006±0.024	0.0345±0.0997	0.000
aVR(ST40)(mv)	-0.0098±0.0242	-0.0110±0.0392	0.627
aVL(ST40)(mv)	0.0038±0.0160	-0.0198±0.0667	0.000
aVF(ST40)(mv)	0.0053±0.0256	0.0308±0.0850	0.000
V1(ST40)(mv)	0.0549±0.0452	0.0645±0.0845	0.046
V2(ST40)(mv)	0.1211±0.081	0.1404±0.1594	0.032
V3(ST40)(mv)	0.0871±0.076	0.1097±0.1649	0.014
V4(ST40)(mv)	0.0476±0.0569	0.0571±0.1324	0.196
V5(ST40)(mv)	0.0207±0.0430	0.0175±0.0896	0.517
V6(ST40)(mv)	0.0081±0.0348	0.0058±0.0604	0.501
I (ST60)(mv)	0.0170±0.02530	-0.0008±0.0521	0.000
II(ST60)(mv)	0.0205±0.0343	0.037±0.0801	0.000
III(ST60)(mv)	0.0025±0.0260	0.0375±0.1031	0.000
aVR(ST60)(mv)	-0.0182±0.0266	-0.0169±0.0431	0.624
aVL(ST60)(mv)	0.0066±0.0182	-0.0185±0.0702	0.000
aVF(ST60)(mv)	0.0106±0.0277	0.0360±0.0879	0.000
V1(ST60)(mv)	0.0626±0.0543	0.0716±0.0920	0.091

V2(ST60)(mv)	0.1551±0.1023	0.1727±0.1721	0.079
V3(ST60)(mv)	0.1194±0.0966	0.1355±0.1757	0.107
V4(ST60)(mv)	0.0742±0.0718	0.0732±0.1428	0.903
V5(ST60)(mv)	0.0387±0.0522	0.0262±0.0986	0.025
V6(ST60)(mv)	0.0208±0.0404	0.0123±0.067	0.028
I (ST80)(mv)	0.0296±0.0319	0.0066±0.0545	0.000
II(ST80)(mv)	0.0353±0.0405	0.0481±0.0867	0.008
III(ST80)(mv)	0.0046±0.0307	0.0412±0.1090	0.000
aVR(ST80)(mv)	-0.0313±0.0321	-0.0258±0.0466	0.047
aVL(ST80)(mv)	0.0115±0.0229	-0.0166±0.0733	0.000
aVF(ST80)(mv)	0.0192±0.0319	0.0432±0.0938	0.000
V1(ST80)(mv)	0.0693±0.0689	0.0782±0.0949	0.122
V2(ST80)(mv)	0.2043±0.1339	0.2156±0.1924	0.330
V3(ST80)(mv)	0.1651±0.1270	0.1650±0.19060	0.997
V4(ST80)(mv)	0.1126±0.0955	0.0892±0.1535	0.010
V5(ST80)(mv)	0.0659±0.0685	0.0349±0.1098	0.000
V6(ST80)(mv)	0.0395±0.0509	0.0182±0.0762	0.000

P value vs control

Supplementary Table 5 ECG features between Cohort 1 and Cohort 2

	Cohort 1	Cohort 2	P value
n	793	90	
Minimum HR(bpm)	70.72±14.92	68.16±14.59	0.122
Maximun HR(bpm)	82.92±23.93	78.90±18.71	0.124
Average HR(bpm)	75.76±15.23	72.67±13.26	0.065
PR(ms)	153.71±25.54	149.08±26.35	0.105
P wave(ms)	107.60±22.04	101.82±21.76	0.019
QRS complex(ms)	104.12±12.94	104.21±8.55	0.949
T wave(ms)	180.21±27.76	180.53±32.32	0.917
QT interval(ms)	388.84±37.71	396.10±37.60	0.084
QTc interval(ms)	433.22±32.94	432.56±28.90	0.854
QRS axis(degree)	39.39±52.32	27.16±48.36	0.034
RV1+SV5 (mv)	0.49±0.55	0.46±0.52	0.552
RV5+SV1 (mv)	2.07±0.88	2.04±0.89	0.789
R-PB(ms)	203.22±26.47	198.41±27.34	0.104
R-P(ms)	142.27±25.62	143.49±25.3	0.669
R-PE(ms)	95.18±16.9	96.59±20.23	0.464
R-Q(ms)	49.49±4.74	49.78±3.55	0.578
R-S(ms)	54.63±10.20	54.43±6.37	0.858
R-TB(ms)	159.15±28.35	165.79±25.77	0.034
R-T(ms)	265.28±39.42	267.72±42.11	0.581
R-TE(ms)	339.35±37.65	346.32±38.25	0.097

Q-P(ms)	93.44±24.94	94.08±24.28	0.817
S-T(ms)	210.65±40.28	213.29±42.26	0.558
Q-PB(ms)	153.65±25.31	148.74±26.27	0.083
S-TE(ms)	284.72±38.97	291.89±39.34	0.099
I (PB)((mv))	0.0597±0.0276	0.0601±0.0313	0.907
II(PB)(mv)	0.1034±0.0454	0.0952±0.0394	0.103
III(PB)(mv)	0.0667±0.0412	0.0583±0.0384	0.065
aVR(PB)(mv)	0.0003±0.0039	0.0004±0.0042	0.745
aVL(PB)(mv)	0.0258±0.0249	0.0279±0.0277	0.457
aVF(PB)(mv)	0.0810±0.0428	0.0734±0.0364	0.107
V1(PB)(mv)	0.0370±0.0278	0.0347±0.029	0.444
V2(PB)(mv)	0.0518±0.0334	0.0479±0.0304	0.293
V3(PB)(mv)	0.0596±0.0293	0.0599±0.0248	0.924
V4(PB)(mv)	0.0600±0.0276	0.0590±0.0225	0.747
V5(PB)(mv)	0.0575±0.0259	0.0596±0.0249	0.472
V6(PB)(mv)	0.0547±0.0253	0.0571±0.0247	0.391
I (PE)(mv)	-0.0005±0.0055	-0.0007±0.0063	0.826
II(PE)(mv)	-0.0007±0.0062	-0.0004±0.0042	0.724
III(PE)(mv)	-0.0107±0.0205	-0.0126±0.0229	0.433
aVR(PE)(mv)	-0.0753±0.0299	-0.0721±0.0279	0.327
aVL(PE)(mv)	-0.0185±0.0224	-0.0168±0.0198	0.479
aVF(PE)(mv)	-0.0015±0.0084	-0.0018±0.0087	0.799
V1(PE)(mv)	-0.0343±0.0314	-0.0378±0.0315	0.325
V2(PE)(mv)	-0.009±0.0197	-0.0072±0.0169	0.41

V3(PE)(mv)	-0.0037±0.0132	-0.0031±0.0171	0.676
V4(PE)(mv)	-0.0022±0.0095	-0.0014±0.0110	0.479
V5(PE)(mv)	-0.0016±0.0081	-0.0012±0.0090	0.709
V6(PE)(mv)	-0.0006±0.005	-0.0007±0.0063	0.932
I (Q)(mv)	-0.0287±0.0653	-0.0198±0.0265	0.197
II(Q)(mv)	-0.0490±0.0800	-0.0734±0.1094	0.009
III(Q)(mv)	-0.1288±0.2324	-0.2156±0.2872	0.001
aVR(Q)(mv)	-0.3334±0.3272	-0.2914±0.2865	0.244
aVL(Q)(mv)	-0.0434±0.0875	-0.0314±0.0587	0.208
aVF(Q)(mv)	-0.0730±0.1304	-0.1221±0.1783	0.013
V1(Q)(mv)	-0.1709±0.3908	-0.2176±0.3967	0.284
V2(Q)(mv)	-0.2123±0.6004	-0.3539±0.6825	0.062
V3(Q)(mv)	-0.1656±0.4971	-0.2291±0.5022	0.252
V4(Q)(mv)	-0.0705±0.2417	-0.0773±0.2584	0.799
V5(Q)(mv)	-0.0478±0.1176	-0.0327±0.0826	0.235
V6(Q)(mv)	-0.0368±0.0561	-0.0342±0.0504	0.682
I (R)(mv)	0.5781±0.3030	0.5574±0.2523	0.472
II(R)(mv)	0.6430±0.3584	0.4989±0.2927	0.000
III(R)(mv)	0.3315±0.3136	0.2482±0.2420	0.003
aVR(R)(mv)	0.0773±0.0931	0.0671±0.0717	0.221
aVL(R)(mv)	0.3620±0.2850	0.3882±0.2403	0.338
aVF(R)(mv)	0.4373±0.3335	0.3141±0.2606	0.000
V1(R)(mv)	0.2027±0.2185	0.1490±0.1767	0.025
V2(R)(mv)	0.5729±0.4567	0.5443±0.5349	0.628

V3(R)(mv)	0.8739±0.6504	0.8157±0.7327	0.472
V4(R)(mv)	1.2815±0.7948	1.1762±0.8433	0.237
V5(R)(mv)	1.2654±0.6961	1.2830±0.6986	0.82
V6(R)(mv)	1.0241±0.5358	1.1129±0.5581	0.138
I (S)(mv)	-0.1022±0.1213	-0.1068±0.1092	0.731
II(S)(mv)	-0.0979±0.1374	-0.0568±0.1180	0.003
III(S)(mv)	-0.1624±0.2443	-0.0911±0.1826	0.001
aVR(S)(mv)	-0.2504±0.3510	-0.2130±0.2945	0.265
aVL(S)(mv)	-0.1422±0.1636	-0.1360±0.1425	0.732
aVF(S)(mv)	-0.0969±0.1512	-0.0507±0.1153	0.005
V1(S)(mv)	-0.8167±0.4327	-0.7694±0.4722	0.331
V2(S)(mv)	-1.0780±0.7173	-0.8996±0.7327	0.026
V3(S)(mv)	-0.8099±0.6281	-0.7889±0.6416	0.764
V4(S)(mv)	-0.5713±0.4723	-0.6044±0.4305	0.525
V5(S)(mv)	-0.3392±0.2998	-0.3671±0.2641	0.398
V6(S)(mv)	-0.1611±0.1895	-0.1759±0.179	0.481
I (R')(mv)	0.0005±0.0112	0.0000±0.0000	0.67
II(R')(mv)	0.0032±0.0222	0.0053±0.0311	0.400
III(R')(mv)	0.0369±0.0968	0.0304±0.0814	0.546
aVR(R')(mv)	0.0245±0.0626	0.0170±0.0458	0.159
aVL(R')(mv)	0.0052±0.0221	0.0014±0.0082	0.001
aVF(R')(mv)	0.0145±0.0573	0.0112±0.0466	0.598
V1(R')(mv)	0.0153±0.1112	0.0026±0.0171	0.277
V2(R')(mv)	0.0024±0.0215	0.001±0.0095	0.550

V3(R')(mv)	0.0027±0.0305	0.0091±0.0864	0.151
V4(R')(mv)	0.0042±0.0497	0.0022±0.0149	0.704
V5(R')(mv)	0.0011±0.0140	0.0000±0.0000	0.459
V6(R')(mv)	0.0003±0.0053	0.0000±0.0000	0.539
I (S')(mv)	-0.0001±0.0014	0.0000±0.0000	0.736
II(S')(mv)	-0.0005±0.0103	0.0000±0.0000	0.635
III(S')(mv)	-0.0056±0.0344	-0.0002±0.0021	0.139
aVR(S')(mv)	-0.0001±0.0014	0.0000±0.0000	0.736
aVL(S')(mv)	-0.0004±0.0077	0.0000±0.0000	0.654
aVF(S')(mv)	-0.0040±0.0340	0.0000±0.0000	0.266
V1(S')(mv)	-0.0013±0.0188	-0.0009±0.0084	0.833
V2(S')(mv)	-0.0044±0.0730	0.0000±0.0000	0.570
V3(S')(mv)	-0.0003±0.0065	-0.0093±0.0885	0.337
V4(S')(mv)	-0.0026±0.0422	-0.0063±0.0601	0.455
V5(S')(mv)	-0.0003±0.0085	0.0000±0.0000	0.736
V6(S')(mv)	-0.0001±0.0036	0.0000±0.0000	0.736
I (TB)(mv)	0.1121±0.0784	0.1000±0.0834	0.169
II(TB)(mv)	0.1333±0.0941	0.0913±0.0837	0.000
III(TB)(mv)	0.0632±0.0761	0.0541±0.0759	0.283
aVR(TB)(mv)	0.0052±0.0219	0.0089±0.0227	0.135
aVL(TB)(mv)	0.0635±0.0699	0.0866±0.1011	0.005
aVF(TB)(mv)	0.0916±0.0796	0.0657±0.0758	0.003
V1(TB)(mv)	0.0696±0.0917	0.0672±0.106	0.822
V2(TB)(mv)	0.3100±0.2258	0.3037±0.2832	0.807

V3(TB)(mv)	0.2779±0.2281	0.2688±0.2578	0.722
V4(TB)(mv)	0.2452±0.2081	0.2082±0.2156	0.112
V5(TB)(mv)	0.1921±0.1599	0.1547±0.1710	0.037
V6(TB)(mv)	0.1528±0.1238	0.1242±0.1363	0.040
I (TE)(mv)	0.1121±0.0784	0.1000±0.0834	0.169
II(TE)(mv)	0.1333±0.0941	0.0913±0.0837	0.000
III(TE)(mv)	0.0632±0.0761	0.0541±0.0759	0.283
aVR(TE)(mv)	0.0052±0.0219	0.0089±0.0227	0.149
aVL(TE)(mv)	0.0635±0.0699	0.0866±0.1011	0.038
aVF(TE)(mv)	0.0916±0.0796	0.0657±0.0758	0.003
V1(TE)(mv)	0.0696±0.0917	0.0672±0.1060	0.822
V2(TE)(mv)	0.3100±0.2258	0.3037±0.2832	0.807
V3(TE)(mv)	0.2779±0.2281	0.2688±0.2578	0.722
V4(TE)(mv)	0.2452±0.2081	0.2082±0.2156	0.112
V5(TE)(mv)	0.1921±0.1599	0.1547±0.1710	0.037
V6(TE)(mv)	0.1528±0.1238	0.1242±0.1363	0.040
I (ST20)(mv)	-0.0028±0.0321	-0.0147±0.0467	0.002
II(ST20)(mv)	0.0127±0.0564	0.0194±0.0480	0.279
III(ST20)(mv)	0.0147±0.0683	0.0327±0.0804	0.021
aVR(ST20)(mv)	-0.0047±0.0305	-0.0020±0.0240	0.425
aVL(ST20)(mv)	-0.0085±0.0446	-0.0230±0.0611	0.031
aVF(ST20)(mv)	0.0131±0.0601	0.0251±0.0608	0.073
V1(ST20)(mv)	0.0458±0.0593	0.0539±0.0979	0.258
V2(ST20)(mv)	0.0952±0.1100	0.0837±0.1180	0.349

V3(ST20)(mv)	0.0659±0.1132	0.0608±0.1190	0.684
V4(ST20)(mv)	0.0288±0.0886	0.0252±0.1036	0.721
V5(ST20)(mv)	0.0044±0.0609	0.0062±0.0731	0.795
V6(ST20)(mv)	-0.0007±0.0434	-0.0042±0.0487	0.474
I (ST40)(mv)	0.0040±0.0345	-0.0080±0.0448	0.003
II(ST40)(mv)	0.0180±0.0563	0.0238±0.0545	0.351
III(ST40)(mv)	0.0133±0.0674	0.0306±0.0858	0.026
aVR(ST40)(mv)	-0.0107±0.0321	-0.0071±0.0251	0.308
aVL(ST40)(mv)	-0.0048±0.0445	-0.0189±0.0618	0.007
aVF(ST40)(mv)	0.0150±0.0591	0.0262±0.0678	0.094
V1(ST40)(mv)	0.0586±0.0659	0.0626±0.0576	0.586
V2(ST40)(mv)	0.1301±0.1211	0.1228±0.1224	0.589
V3(ST40)(mv)	0.0966±0.1222	0.0980±0.1269	0.918
V4(ST40)(mv)	0.0516±0.0952	0.0522±0.1096	0.954
V5(ST40)(mv)	0.0187±0.0656	0.0248±0.0786	0.414
V6(ST40)(mv)	0.0073±0.0470	0.0054±0.0520	0.723
I (ST60)(mv)	0.0106±0.0389	-0.0016±0.0475	0.006
II(ST60)(mv)	0.0271±0.0586	0.031±0.0621	0.553
III(ST60)(mv)	0.0158±0.0692	0.0319±0.0941	0.045
aVR(ST60)(mv)	-0.0181±0.0353	-0.0134±0.028	0.224
aVL(ST60)(mv)	-0.0028±0.0469	-0.0157±0.067	0.019
aVF(ST60)(mv)	0.0204±0.0607	0.0302±0.0755	0.159
V1(ST60)(mv)	0.0662±0.0741	0.0692±0.0616	0.707
V2(ST60)(mv)	0.1634±0.1369	0.1561±0.1355	0.633

V3(ST60)(mv)	0.1258±0.1355	0.1300±0.1431	0.784
V4(ST60)(mv)	0.0736±0.1065	0.0756±0.1205	0.870
V5(ST60)(mv)	0.0325±0.0743	0.0409±0.0882	0.320
V6(ST60)(mv)	0.0173±0.053	0.0167±0.0581	0.922
I (ST80)(mv)	0.0211±0.0432	0.0081±0.0533	0.009
II(ST80)(mv)	0.0408±0.0639	0.0402±0.0713	0.931
III(ST80)(mv)	0.0190±0.0731	0.0316±0.1057	0.142
aVR(ST80)(mv)	-0.0297±0.0396	-0.0228±0.0326	0.113
aVL(ST80)(mv)	0.0007±0.0495	-0.0113±0.0751	0.040
aVF(ST80)(mv)	0.0289±0.0646	0.0347±0.0850	0.439
V1(ST80)(mv)	0.0727±0.0818	0.0766±0.0747	0.668
V2(ST80)(mv)	0.2096±0.1618	0.2054±0.1598	0.818
V3(ST80)(mv)	0.1644±0.1562	0.1708±0.1670	0.716
V4(ST80)(mv)	0.1024±0.1226	0.1040±0.1378	0.910
V5(ST80)(mv)	0.0517±0.0882	0.0614±0.1032	0.327
V6(ST80)(mv)	0.0304±0.0632	0.0310±0.0696	0.927

P value vs Cohort 1

Supplementary Table 6 ECG features among different locations of culprit vessels

	Control	LAD	RCA	LCX	P value
n	506	194	128	55	
Minimum HR(bpm)	69.45±12.65	72.65±18.03	71.01±17.08	70.71±15.82	0.081
Maximun HR(bpm)	78.72±20.03	88.62±28.14	86.75±24.9	85.93±24.93	0.000
Average HR(bpm)	73.24±12.57	79.46±17.55	77.68±17.90	76.38±16.05	0.000
PR(ms)	151.72±24.46	154.25±26.72	157.17±27.93	154.49±26.39	0.156
P wave(ms)	108.66±22.01	105.11±23.00	103.38±22.29	106.96±17.21	0.052
QRS complex(ms)	102.87±11.02	107.12±12.53	105.95±17.80	101.00±8.17	0.000
T wave(ms)	183.65±21.66	165.42±33.14	185.33±31.13	189.27±37.21	0.000
QT interval(ms)	386.41±32.35	390.19±45.15	397.05±39.0	399.31±47.72	0.006
QTc interval(ms)	424.23±26.77	443.71±36.61	446.90±34.07	446.05±36.00	0.000
QRS axis(degree)	43.16±37.62	45.5±70.59	10.09±55.94	31.31±58.99	0.000
RV1+SV5 (mv)	0.54±0.54	0.37±0.54	0.41±0.54	0.60±0.53	0.000
RV5+SV1 (mv)	2.29±0.74	1.60±0.90	1.92±0.98	1.96±0.98	0.000
R-PB(ms)	201.11±25.66	204.91±26.85	205.72±29.06	202.95±27.53	0.187
R-P(ms)	137.56±21.21	148.43±27.74	152.14±30.87	142.96±30.02	0.000
R-PE(ms)	92.29±12.81	99.19±17.34	101.67±24.71	94.85±24.10	0.000
R-Q(ms)	49.39±3.97	50.48±5.07	49.33±6.05	47.78±4.263	0.001
R-S(ms)	53.48±8.25	56.64±9.61	56.62±15.31	53.22±5.78	0.000
R-TB(ms)	153.37±23.19	174.29±29.48	162.39±31.88	162.25±35.46	0.000
R-T(ms)	264.55±39.61	259.73±36.83	275.00±43.11	273±37.78	0.003

R-TE(ms)	337.02±31.99	339.71±45.48	347.72±39.79	351.53±47.18	0.003
Q-P(ms)	88.43±20.19	99.19±27.63	104.52±29.79	94.42±28.57	0.000
S-T(ms)	211.07±39.71	203.09±37.98	218.38±45.78	219.78±38.46	0.002
Q-PB(ms)	151.70±24.44	154.35±26.23	156.70±27.42	153.95±26.37	0.201
S-TE(ms)	283.54±32.67	283.07±47.21	291.10±43.10	298.31±47.81	0.014
I (PB)((mv))	0.0579±0.0262	0.0647±0.0302	0.0566±0.0297	0.0667±0.0280	0.004
II(PB)(mv)	0.1054±0.0432	0.1009±0.0467	0.0886±0.0454	0.1145±0.0445	0.000
III(PB)(mv)	0.0695±0.0423	0.0624±0.0395	0.0549±0.0342	0.0705±0.0430	0.002
aVR(PB)(mv)	0.0001±0.0013	0.0005±0.0051	0.0012±0.0075	0.0000±0.0000	0.027
aVL(PB)(mv)	0.0241±0.0238	0.0312±0.0277	0.0254±0.0257	0.0269±0.0254	0.009
aVF(PB)(mv)	0.0833±0.0426	0.0781±0.0413	0.0678±0.0391	0.0885±0.0424	0.001
V1(PB)(mv)	0.0397±0.0264	0.0353±0.0304	0.0281±0.0280	0.0360±0.0275	0.000
V2(PB)(mv)	0.0561±0.0281	0.0469±0.0410	0.0382±0.0336	0.0545±0.0340	0.000
V3(PB)(mv)	0.0639±0.0257	0.0557±0.0322	0.0461±0.0303	0.0653±0.0287	0.000
V4(PB)(mv)	0.0638±0.0239	0.0571±0.0304	0.0468±0.0278	0.0638±0.0307	0.000
V5(PB)(mv)	0.0607±0.0228	0.0553±0.0291	0.0466±0.0268	0.0647±0.0280	0.000
V6(PB)(mv)	0.0565±0.0222	0.0541±0.0295	0.0457±0.0269	0.0647±0.0247	0.000
I (PE)(mv)	-0.0001±0.001	-0.0015±0.0099	-0.0011±0.0074	0.0000±0.0000	0.014
II(PE)(mv)	-0.0004±0.0033	-0.0006±0.0050	-0.0020±0.0123	0.0000±0.0000	0.041
III(PE)(mv)	-0.0096±0.0189	-0.0148±0.0240	-0.0113±0.0229	-0.0084±0.0176	0.023
aVR(PE)(mv)	-0.0757±0.0270	-0.0756±0.0326	-0.0669±0.0334	-0.0858±0.0289	0.001
aVL(PE)(mv)	-0.02±0.0229	-0.0162±0.0214	-0.0155±0.0188	-0.0171±0.0233	0.068
aVF(PE)(mv)	-0.0012±0.0066	-0.0022±0.0093	-0.0025±0.0132	-0.0005±0.0040	0.234
V1(PE)(mv)	-0.0294±0.0289	-0.0431±0.0332	-0.0366±0.0314	-0.0489±0.0349	0.000

V2(PE)(mv)	-0.0065±0.0165	-0.011±0.0218	-0.0134±0.0242	-0.0115±0.0192	0.001
V3(PE)(mv)	-0.0019±0.0086	-0.0057±0.0174	-0.0079±0.0208	-0.0036±0.0123	0.000
V4(PE)(mv)	-0.0009±0.0058	-0.0041±0.0131	-0.0037±0.0140	-0.0025±0.0094	0.000
V5(PE)(mv)	-0.0008±0.0050	-0.0031±0.0122	-0.0022±0.0109	-0.0011±0.0056	0.006
V6(PE)(mv)	-0.0005±0.0030	-0.0011±0.0076	-0.0007±0.0059	0.0000±0.0000	0.358
I (Q)(mv)	-0.0264±0.0320	-0.0295±0.0554	-0.0295±0.1328	-0.0313±0.0396	0.884
II(Q)(mv)	-0.0281±0.0397	-0.0488±0.0862	-0.1350±0.1316	-0.0818±0.0983	0.000
III(Q)(mv)	-0.0562±0.1088	-0.1246±0.2055	-0.4503±0.3654	-0.2051±0.2542	0.000
aVR(Q)(mv)	-0.3847±0.3407	-0.2958±0.2670	-0.2194±0.2876	-0.1905±0.3033	0.000
aVL(Q)(mv)	-0.0431±0.0698	-0.0543±0.0873	-0.0244±0.1333	-0.0316±0.0455	0.015
aVF(Q)(mv)	-0.0335±0.0602	-0.0682±0.1162	-0.2509±0.2149	-0.1200±0.1521	0.000
V1(Q)(mv)	-0.0463±0.2050	-0.5989±0.5442	-0.0904±0.2522	-0.0720±0.2696	0.000
V2(Q)(mv)	-0.0142±0.1414	-0.9570±0.9577	-0.0266±0.1779	-0.0727±0.2920	0.000
V3(Q)(mv)	-0.0134±0.1467	-0.7169±0.8223	-0.0255±0.0781	-0.0516±0.2120	0.000
V4(Q)(mv)	-0.0125±0.0628	-0.2659±0.4544	-0.0248±0.0627	-0.0318±0.0846	0.000
V5(Q)(mv)	-0.0234±0.0417	-0.1019±0.2056	-0.0502±0.1048	-0.0513±0.0701	0.000
V6(Q)(mv)	-0.0302±0.0364	-0.0339±0.0711	-0.0537±0.0767	-0.0638±0.0649	0.000
I (R)(mv)	0.6059±0.2791	0.4321±0.2679	0.6615±0.3333	0.6096±0.3230	0.000
II(R)(mv)	0.7712±0.3212	0.4670±0.3056	0.3829±0.3063	0.4529±0.2783	0.000
III(R)(mv)	0.3672±0.3255	0.2824±0.2646	0.2473±0.3021	0.2356±0.2191	0.000
aVR(R)(mv)	0.0728±0.0847	0.0821±0.0983	0.0829±0.1096	0.0715±0.0742	0.503
aVL(R)(mv)	0.3281±0.2544	0.3073±0.2483	0.5575±0.3223	0.4540±0.3114	0.000
aVF(R)(mv)	0.519±0.3330	0.3375±0.2770	0.2448±0.28693	0.2838±0.2311	0.000
V1(R)(mv)	0.2283±0.1864	0.0832±0.2301	0.2059±0.22043	0.2935±0.2482	0.000

V2(R)(mv)	0.6522±0.3490	0.1201±0.2501	0.782±0.56957	0.9069±0.6266	0.000
V3(R)(mv)	1.0015±0.50477	0.2249±0.4389	1.1047±0.74968	1.3569±0.8198	0.000
V4(R)(mv)	1.5120±0.6068	0.4464±0.6157	1.4534±0.90145	1.5344±0.7794	0.000
V5(R)(mv)	1.4901±0.56115	0.6783±0.6283	1.2898±0.80291	1.2407±0.5710	0.000
V6(R)(mv)	1.2020±0.45286	0.6991±0.5085	0.9187±0.62418	0.9244±0.5152	0.000
I (S)(mv)	-0.1014±0.1142	-0.0961±0.1177	-0.1083±0.1433	-0.1236±0.1229	0.461
II(S)(mv)	-0.0929±0.1345	-0.1088±0.1466	-0.0716±0.1241	-0.0982±0.1353	0.120
III(S)(mv)	-0.1738±0.2358	-0.1604±0.2459	-0.0607±0.1760	-0.1855±0.3222	0.000
aVR(S)(mv)	-0.2839±0.3875	-0.1215±0.2244	-0.2610±0.2978	-0.3118±0.2921	0.000
aVL(S)(mv)	-0.1518±0.1614	-0.1132±0.1507	-0.1451±0.1785	-0.1385±0.1502	0.044
aVF(S)(mv)	-0.0979±0.1453	-0.1101±0.1674	-0.0405±0.1038	-0.0958±0.1718	0.000
V1(S)(mv)	-0.8266±0.3991	-0.9065±0.4874	-0.633±0.4038	-0.7596±0.5311	0.000
V2(S)(mv)	-1.2228±0.6015	-0.6809±0.9243	-1.0057±0.5962	-1.0233±0.6578	0.000
V3(S)(mv)	-0.9097±0.6044	-0.5382±0.7115	-0.7994±0.5016	-0.8402±0.5363	0.000
V4(S)(mv)	-0.6115±0.4739	-0.5070±0.5042	-0.5337±0.4033	-0.5705±0.3928	0.042
V5(S)(mv)	-0.3514±0.3037	-0.3613±0.2979	-0.2909±0.2793	-0.3071±0.2476	0.116
V6(S)(mv)	-0.1760±0.1940	-0.1498±0.1830	-0.1280±0.1770	-0.1658±0.1720	0.050
I (R')(mv)	0.0001±0.0022	00.0000±0.0000	0.0027±0.0276	0.0000±0.0000	0.075
II(R')(mv)	0.0019±0.0130	0.0064±0.0366	0.0027±0.0174	0.0075±0.0415	0.072
III(R')(mv)	0.0485±0.1103	0.0254±0.0743	0.0070±0.0477	0.0296±0.0754	0.000
aVR(R')(mv)	0.0174±0.0507	0.0212±0.058	0.0402±0.0807	0.0531±0.0882	0.000
aVL(R')(mv)	0.0061±0.0225	0.0035±0.0192	0.0025±0.0208	0.0027±0.0145	0.186
aVF(R')(mv)	0.0150±0.0588	0.0192±0.0653	0.0048±0.0277	0.0105±0.0445	0.139
V1(R')(mv)	0.0188±0.1287	0.0062±0.0613	0.0081±0.0521	0.0111±0.0823	0.469

V2(R')(mv)	0.0016±0.0179	0.0008±0.0108	0.0067±0.0375	0.0024±0.0123	0.058
V3(R')(mv)	0.0028±0.0325	0.0042±0.0589	0.0028±0.023	0.0067±0.0499	0.899
V4(R')(mv)	0.0017±0.0146	0.0076±0.0718	0.0076±0.0796	0.0045±0.0337	0.378
V5(R')(mv)	0.0012±0.0139	0.0014±0.0175	0.0000±0.0000	0.0000±0.0000	0.724
V6(R')(mv)	0.0002±0.0028	0.0008±0.0096	0.0000±0.0000	0.0000±0.0000	0.398
I (S')(mv)	-0.0001±0.0018	0.0000±0.0000	0.0000±0.0000	0.0000±0.0000	0.863
II(S')(mv)	0.0000±0.0000	-0.0021±0.0208	0.0000±0.0000	0.0000±0.0000	0.069
III(S')(mv)	-0.0062±0.0349	-0.0052±0.0348	-0.0023±0.0265	0.0000±0.0000	0.412
aVR(S')(mv)	-0.0001±0.0018	0.0000±0.0000	0.0000±0.0000	0.0000±0.0000	0.863
aVL(S')(mv)	-0.0005±0.0096	0.0000±0.0000	0.0000±0.0000	-0.0005±0.0041	0.797
aVF(S')(mv)	-0.0024±0.0246	-0.0098±0.0556	0.0000±0.0000	-0.0007±0.0054	0.019
V1(S')(mv)	-0.0022±0.0238	0.0000±0.0000	0.0000±0.0000	0.0000±0.0000	0.355
V2(S')(mv)	-0.0013±0.0194	0.0000±0.0000	-0.0057±0.0378	-0.0378±0.2645	0.002
V3(S')(mv)	-0.0002±0.004	-0.0043±0.0603	-0.0013±0.0141	0.0000±0.0000	0.393
V4(S')(mv)	-0.0003±0.0058	-0.0117±0.0917	-0.0021±0.0239	0.0000±0.0000	0.021
V5(S')(mv)	0.0000±0.0000	-0.0012±0.0172	0.0000±0.0000	0.0000±0.0000	0.315
V6(S')(mv)	0.0000±0.0000	-0.0005±0.0072	0.0000±0.0000	0.0000±0.0000	0.315
I (TB)(mv)	0.1331±0.0704	0.0509±0.0657	0.1242±0.0815	0.0869±0.0766	0.000
II(TB)(mv)	0.1600±0.0846	0.1131±0.0933	0.0580±0.0793	0.0649±0.0723	0.000
III(TB)(mv)	0.0566±0.0630	0.0992±0.0945	0.0367±0.0762	0.0440±0.0689	0.000
aVR(TB)(mv)	0.0011±0.0089	0.0113±0.0323	0.0120±0.0305	0.0116±0.0286	0.000
aVL(TB)(mv)	0.0607±0.0586	0.0272±0.0514	0.1364±0.0984	0.0853±0.0844	0.000
aVF(TB)(mv)	0.0999±0.0729	0.1023±0.0885	0.0421±0.0740	0.0498±0.0658	0.000
V1(TB)(mv)	0.0596±0.0916	0.0575±0.0745	0.0937±0.0870	0.1438±0.1309	0.000

V2(TB)(mv)	0.3247±0.2075	0.1548±0.1599	0.4104±0.2529	0.4780±0.3073	0.000
V3(TB)(mv)	0.3171±0.2112	0.1087±0.1493	0.3317±0.2552	0.3744±0.2981	0.000
V4(TB)(mv)	0.3108±0.1970	0.0811±0.1277	0.2141±0.2074	0.2320±0.2283	0.000
V5(TB)(mv)	0.2583±0.1540	0.0710±0.1027	0.1207±0.1283	0.1155±0.1230	0.000
V6(TB)(mv)	0.2045±0.1155	0.0752±0.0933	0.0812±0.0995	0.0709±0.1089	0.000
I (TE)(mv)	0.1331±0.0704	0.0509±0.0657	0.1242±0.0815	0.0869±0.0766	0.000
II(TE)(mv)	0.1600±0.0846	0.1131±0.0933	0.0580±0.0793	0.0649±0.0723	0.000
III(TE)(mv)	0.0566±0.0630	0.0992±0.0945	0.0367±0.0762	0.0440±0.0689	0.000
aVR(TE)(mv)	0.0011±0.0089	0.0113±0.0323	0.0120±0.0305	0.0116±0.0286	0.000
aVL(TE)(mv)	0.0607±0.0586	0.0272±0.0514	0.1364±0.0984	0.0853±0.0844	0.000
aVF(TE)(mv)	0.0999±0.0729	0.1023±0.0885	0.0421±0.0740	0.0498±0.0658	0.000
V1(TE)(mv)	0.0596±0.0916	0.0575±0.0745	0.0937±0.0870	0.1438±0.1309	0.000
V2(TE)(mv)	0.3247±0.2075	0.1548±0.1599	0.4104±0.2529	0.4780±0.3073	0.000
V3(TE)(mv)	0.3171±0.2112	0.1087±0.1493	0.3317±0.2552	0.3744±0.2981	0.000
V4(TE)(mv)	0.3108±0.1970	0.0811±0.1277	0.2141±0.2074	0.2320±0.2283	0.000
V5(TE)(mv)	0.2583±0.1540	0.0710±0.1027	0.1207±0.1283	0.1155±0.1230	0.000
V6(TE)(mv)	0.2045±0.1155	0.0752±0.0933	0.0812±0.0995	0.0709±0.1089	0.000
I (ST20)(mv)	0.0018±0.0199	0.0021±0.0401	-0.0309±0.0447	-0.016±0.0501	0.000
II(ST20)(mv)	0.0057±0.0295	0.0043±0.0522	0.0525±0.1033	0.0258±0.0546	0.000
III(ST20)(mv)	0.0025±0.0226	0.0020±0.0647	0.0832±0.1308	0.0416±0.0754	0.000
aVR(ST20)(mv)	-0.0036±0.0222	-0.0027±0.0332	-0.0102±0.0450	-0.004±0.0342	0.125
aVL(ST20)(mv)	-0.0005±0.0147	0.0007±0.0467	-0.0559±0.0825	-0.0276±0.0567	0.000
aVF(ST20)(mv)	0.0038±0.0243	0.0023±0.0545	0.0662±0.1155	0.0324±0.0601	0.000
V1(ST20)(mv)	0.0413±0.0374	0.0774±0.0841	0.0330±0.0941	0.0191±0.0582	0.000

V2(ST20)(mv)	0.0854±0.0657	0.1855±0.1435	0.0172±0.0933	0.0298±0.1309	0.000
V3(ST20)(mv)	0.0555±0.0622	0.1516±0.1567	0.0039±0.1113	-0.004±0.1294	0.000
V4(ST20)(mv)	0.0235±0.0490	0.0893±0.1280	-0.0213±0.0852	-0.0247±0.1124	0.000
V5(ST20)(mv)	0.0037±0.0389	0.0304±0.0870	-0.0220±0.0688	-0.0162±0.0799	0.000
V6(ST20)(mv)	-0.0013±0.0319	0.0062±0.0576	-0.0095±0.0461	-0.0051±0.0691	0.014
I (ST40)(mv)	0.0092±0.0217	0.0078±0.0401	-0.0258±0.0493	-0.0082±0.0512	0.000
II(ST40)(mv)	0.0107±0.0310	0.0104±0.0537	0.0568±0.1025	0.0304±0.0551	0.000
III(ST40)(mv)	0.0006±0.0240	0.0023±0.0627	0.0818±0.1316	0.0376±0.0732	0.000
aVR(ST40)(mv)	-0.0098±0.0242	-0.0086±0.0347	-0.0148±0.0459	-0.0102±0.0375	0.343
aVL(ST40)(mv)	0.0038±0.016	0.0024±0.0442	-0.0524±0.0843	-0.0222±0.0548	0.000
aVF(ST40)(mv)	0.0053±0.0256	0.0060±0.0540	0.0673±0.1151	0.0335±0.0582	0.000
V1(ST40)(mv)	0.0549±0.0452	0.0853±0.0695	0.0470±0.1037	0.0320±0.0621	0.000
V2(ST40)(mv)	0.1211±0.0810	0.2188±0.1528	0.0531±0.1117	0.0671±0.1367	0.000
V3(ST40)(mv)	0.0871±0.0760	0.1821±0.1654	0.0348±0.1219	0.0284±0.1333	0.000
V4(ST40)(mv)	0.0476±0.0569	0.1116±0.1353	0.0003±0.0941	-0.0035±0.1169	0.000
V5(ST40)(mv)	0.0207±0.0430	0.0425±0.0943	-0.0115±0.0738	-0.0035±0.0815	0.000
V6(ST40)(mv)	0.0081±0.0348	0.0123±0.0623	-0.003±0.0518	0.0033±0.0699	0.032
I (ST60)(mv)	0.017±0.0253	0.0121±0.0449	-0.0202±0.0557	-0.0013±0.0537	0.000
II(ST60)(mv)	0.0205±0.0343	0.0191±0.0566	0.0633±0.1071	0.0389±0.0586	0.000
III(ST60)(mv)	0.0025±0.0260	0.0065±0.0638	0.0837±0.1390	0.0387±0.0739	0.000
aVR(ST60)(mv)	-0.0182±0.0266	-0.0147±0.0387	-0.0202±0.0492	-0.0175±0.0429	0.531
aVL(ST60)(mv)	0.0066±0.0182	0.0028±0.0465	-0.0509±0.0907	-0.0184±0.0555	0.000
aVF(ST60)(mv)	0.0106±0.0277	0.012±0.0554	0.0717±0.1204	0.0375±0.0603	0.000
V1(ST60)(mv)	0.0626±0.0543	0.0913±0.0777	0.0552±0.1121	0.0407±0.0699	0.000

V2(ST60)(mv)	0.1551±0.1023	0.2468±0.1653	0.0882±0.1365	0.1078±0.1541	0.000
V3(ST60)(mv)	0.1194±0.0966	0.2044±0.1764	0.0636±0.1407	0.0600±0.1486	0.000
V4(ST60)(mv)	0.0742±0.0718	0.1256±0.1470	0.0187±0.1086	0.0156±0.1294	0.000
V5(ST60)(mv)	0.0387±0.0522	0.0504±0.1043	-0.0024±0.0825	0.0073±0.0917	0.000
V6(ST60)(mv)	0.0208±0.0404	0.0185±0.0689	0.0034±0.0590	0.0109±0.0760	0.008
I (ST80)(mv)	0.0296±0.0319	0.0172±0.0497	-0.0079±0.0568	0.0029±0.0580	0.000
II(ST80)(mv)	0.0353±0.0405	0.0309±0.0627	0.0734±0.1142	0.0500±0.0710	0.000
III(ST80)(mv)	0.0046±0.0307	0.0135±0.0685	0.081±0.1478	0.0464±0.0898	0.000
aVR(ST80)(mv)	-0.0313±0.0321	-0.0227±0.0435	-0.0309±0.0513	-0.0247±0.0452	0.049
aVL(ST80)(mv)	0.0115±0.0229	0.0022±0.0497	-0.0433±0.0953	-0.0205±0.0649	0.000
aVF(ST80)(mv)	0.0192±0.0319	0.0212±0.06	0.0751±0.1283	0.0469±0.0746	0.000
V1(ST80)(mv)	0.0693±0.0689	0.0978±0.0899	0.059±0.1025	0.0538±0.0788	0.000
V2(ST80)(mv)	0.2043±0.1339	0.2796±0.1817	0.1427±0.1646	0.1596±0.2136	0.000
V3(ST80)(mv)	0.1651±0.127	0.224±0.1888	0.1054±0.1619	0.0958±0.1950	0.000

LAD left anterior descending artery, *RCA* right coronary artery, *LCX* left circumflex artery. *P* value vs control

Supplementary Table 7 Sample size

Sample size	Cohort 1	Cohort 1
STEMI	315	62
LAD	163	31
RCA	107	21
LCX	45	10
control	478	28
Total	793	90

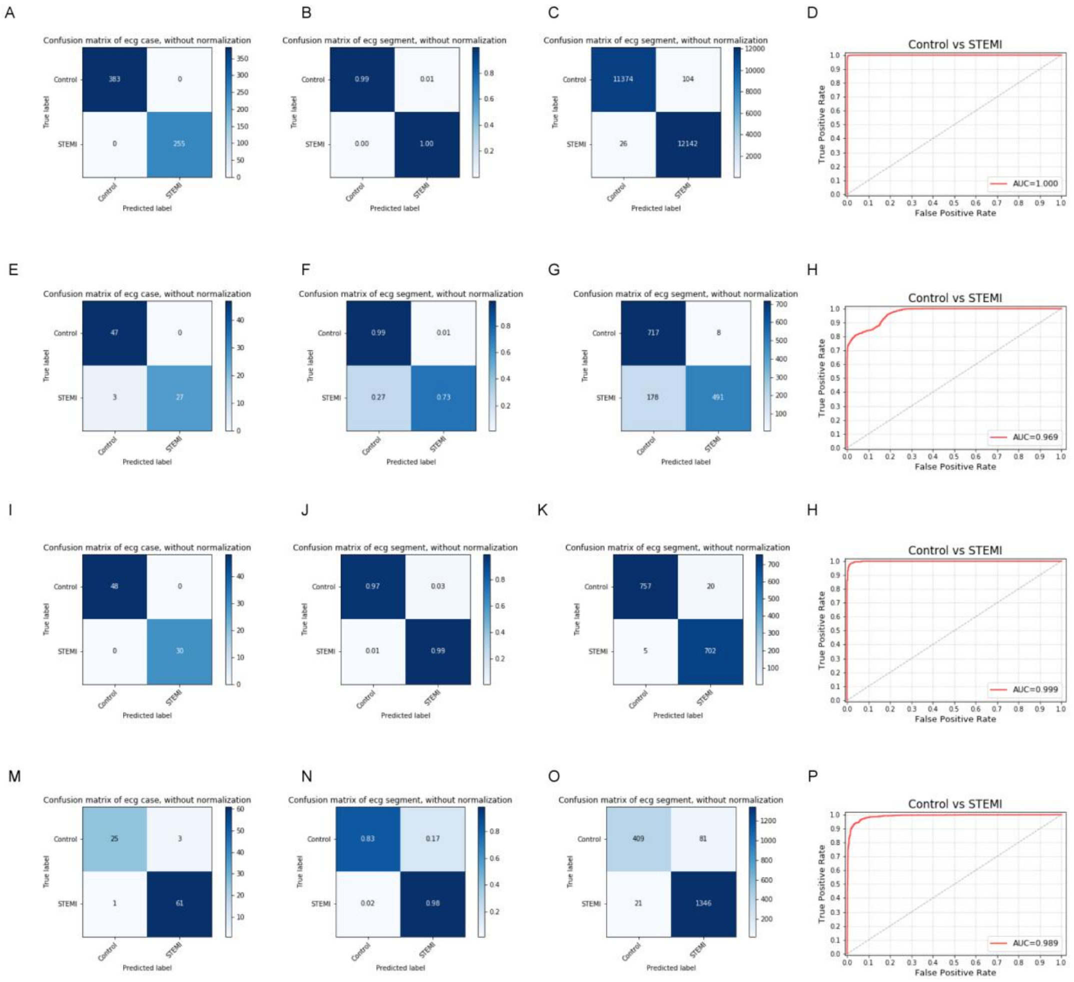

Supplementary Fig. 4 Diagnostic performance of CNN-LSTM to predict Control and STEMI. (A, B, and C) Confusion matrices (cases, proportion, and segments) for the discrimination of Control and STEMI in training dataset. (D) ROC curve of CNN-LSTM in the training dataset. (E, F and G) Confusion matrices (cases, proportion, and segments) in the validation dataset. (H) ROC curve in the validation dataset. (I, J, and K) Confusion matrices (cases, proportion, and segments) in Test 1 dataset. (L) ROC curve in Test 1 dataset. (M, N, and O) Confusion matrices (cases, proportion, and segments) in Test 2 dataset. (P) ROC curve in Test 2 dataset.

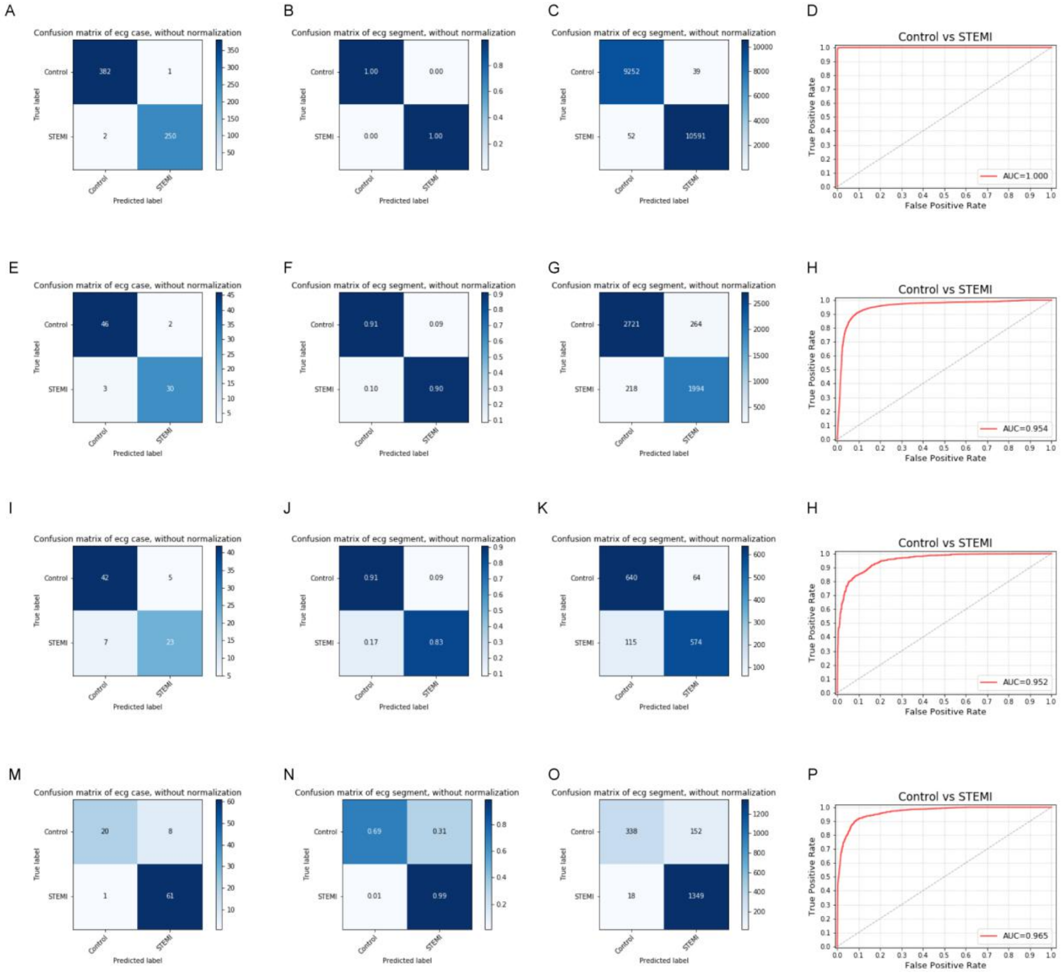

Supplementary Fig. 5 Diagnostic performance of CNN to predict Control and STEMI. (A, B, and C) Confusion matrices (cases, proportion, and segments) for the discrimination of Control and STEMI in training dataset. (D) ROC curve of CNN in the training dataset. (E, F, and G) Confusion matrices (cases, proportion, and segments) in the validation dataset. (H) ROC curve in the validation dataset. (I, ,J and J) Confusion matrices (cases, proportion, and segments) in Test 1 dataset. (L) ROC curve in Test 1 dataset. (M, N and O) Confusion matrices (cases, proportion, and segments) in Test 2 dataset. (P) ROC curve in Test 2 dataset.

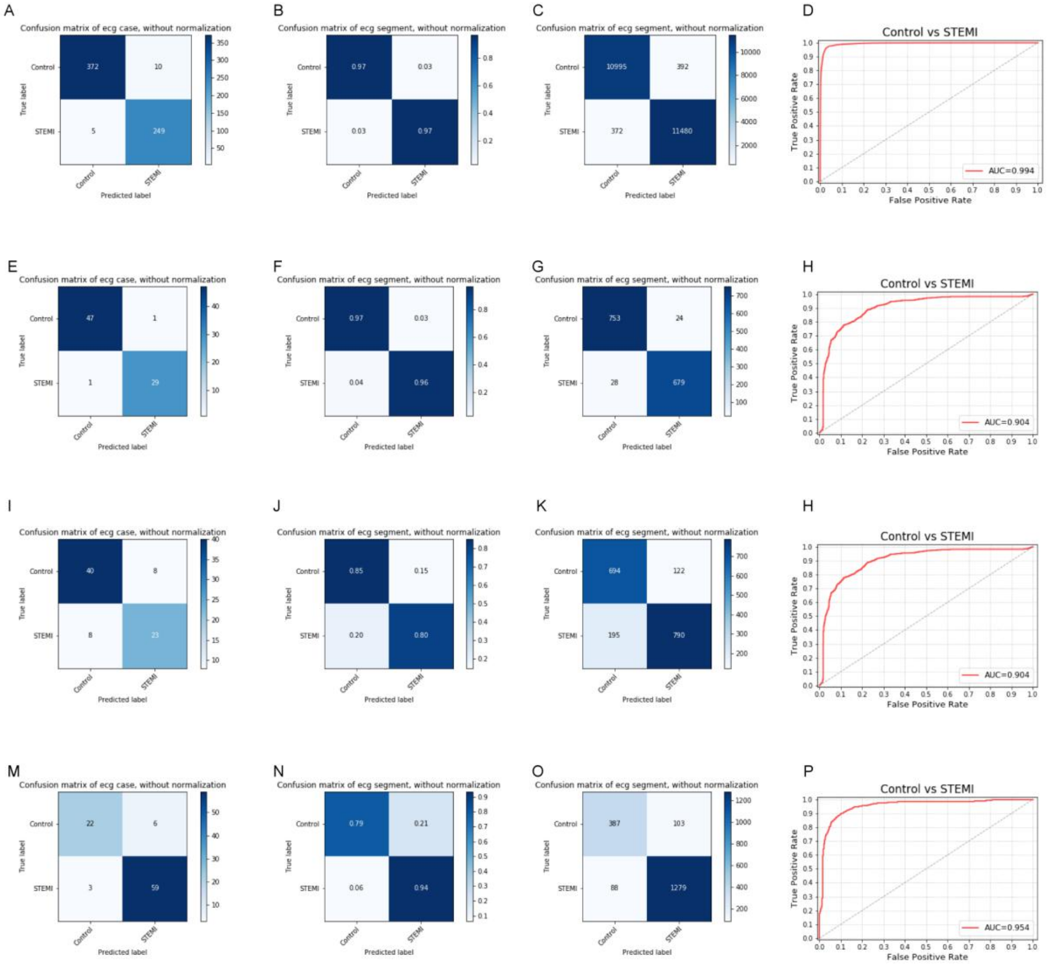

Supplementary Fig. 6 Diagnostic performance of LSTM to predict Control and STEMI. (A, B, and C) Confusion matrices (cases, proportion, and segments) for the discrimination of Control and STEMI in training dataset. (D) ROC curve of LSTM in the training dataset. (E, F, and G) Confusion matrices (cases, proportion, and segments) in the validation dataset. (H) ROC curve in the validation dataset. (I, J, and K) Confusion matrices (cases, proportion, and segments) in Test 1 dataset. (L) ROC curve in Test 1 dataset. (M, N, and O) Confusion matrices (cases, proportion, and segments) in Test 2 dataset. (P) ROC curve in Test 2 dataset.

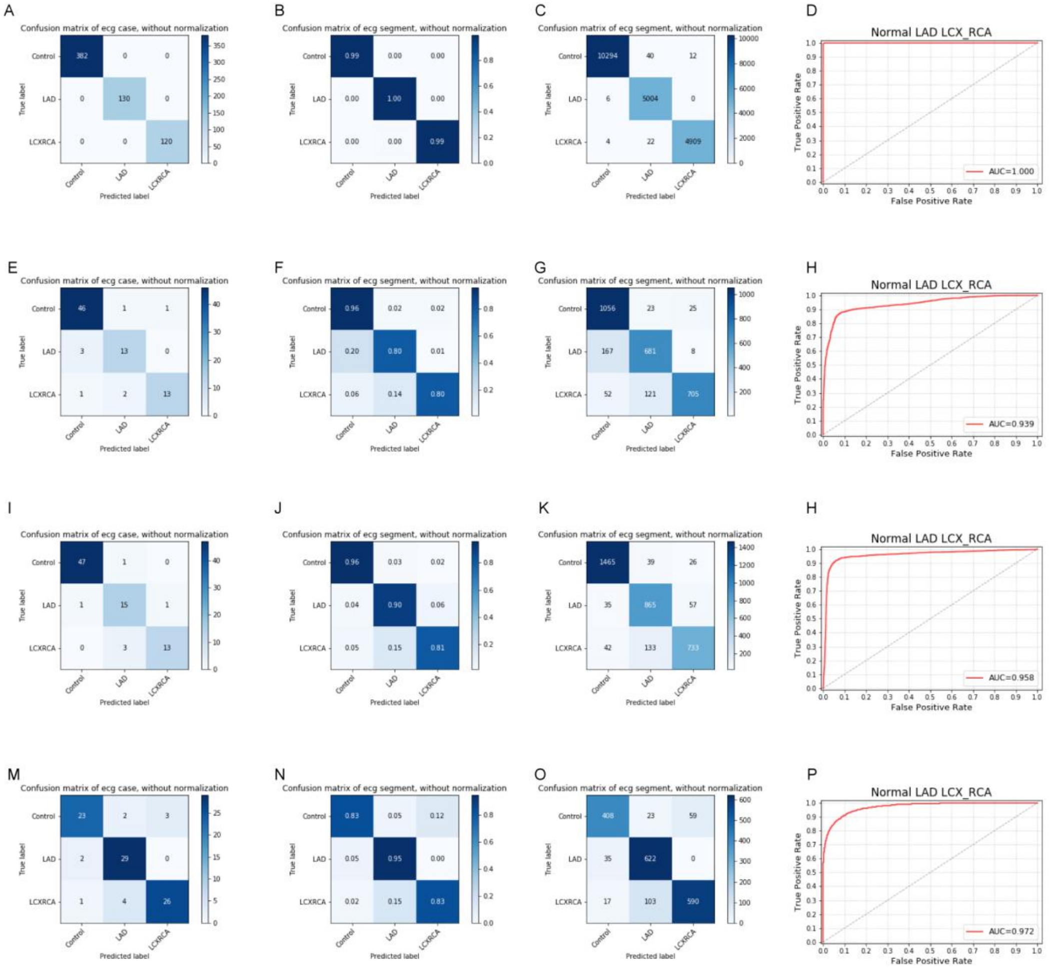

Supplementary Fig. 7 Diagnostic performance of CNN-LSTM to predict Control, LAD, and RCA-LCX. (A, B, and C) Confusion matrices (cases, proportion, and segments) for the discrimination of Control, LAD, and RCA-LCX in training dataset. (D) ROC curve of CNN-LSTM in the training dataset. (E, F, and G) Confusion matrices (cases, proportion, and segments) in the validation dataset. (H) ROC curve in the validation dataset. (I, J, and K) Confusion matrices (cases, proportion, and segments) in Test 1 dataset. (L) ROC curve in Test 1 dataset. (M, N, and O) Confusion matrices (cases, proportion, and segments) in Test 2 dataset. (P) ROC curve in Test 2 dataset.

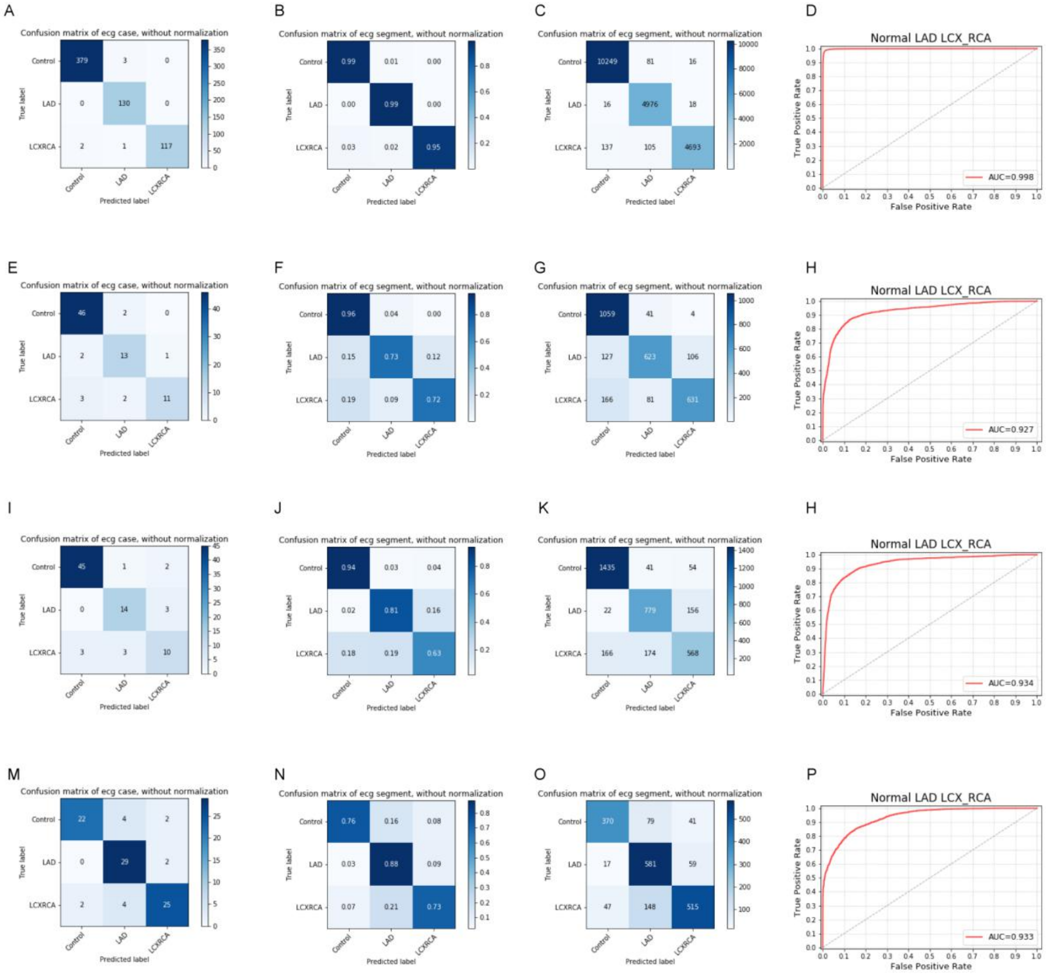

Supplementary Fig. 8 Diagnostic performance of CNN to predict Control, LAD, and RCA-LCX. (A, B, and C) Confusion matrices (cases, proportion, and segments) for the discrimination of Control, LAD, and RCA-LCX in training dataset. (D) ROC curve of CNN in the training dataset. (E, F, and G) Confusion matrices (cases, proportion, and segments) in the validation dataset. (H) ROC curve in the validation dataset. (I, J, and K) Confusion matrices (cases, proportion, and segments) in Test 1 dataset. (L) ROC curve in Test 1 dataset. (M, N, and O) Confusion matrices (cases, proportion, and segments) in Test 2 dataset. (P) ROC curve in Test 2 dataset.

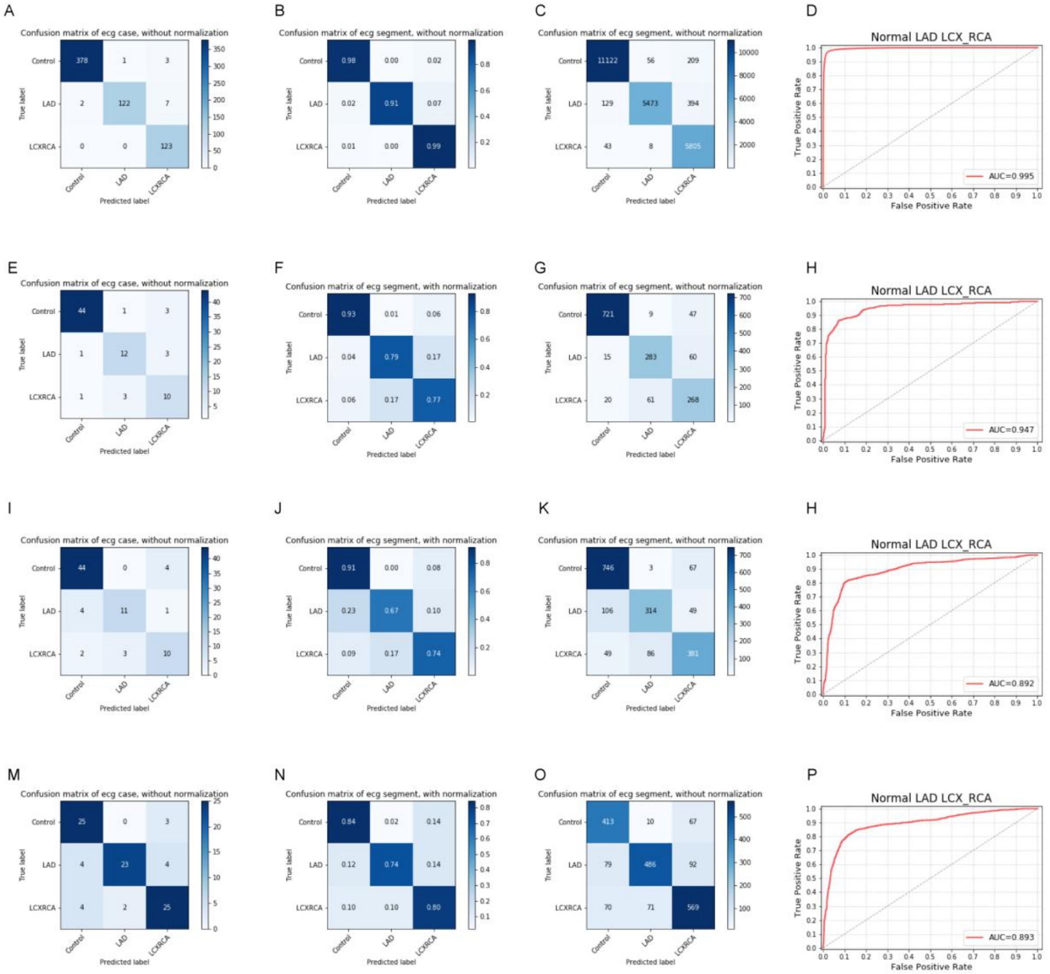

Supplementary Fig. 9 Diagnostic performance of LSTM to predict Control, LAD, and RCA-LCX. (A, B, and C) Confusion matrices (cases, proportion, and segments) for the discrimination of Control, LAD, and RCA-LCX in training dataset. (D) ROC curve of LSTM in the training dataset. (E, F, and G) Confusion matrices (cases, proportion, and segments) in the validation dataset. (H) ROC curve in the validation dataset. (I, J, and K) Confusion matrices (cases, proportion, and segments) in Test 1 dataset. (L) ROC curve in Test 1 dataset. (M, N, and O) Confusion matrices (cases, proportion, and segments) in Test 2 dataset. (P) ROC curve in Test 2 dataset.

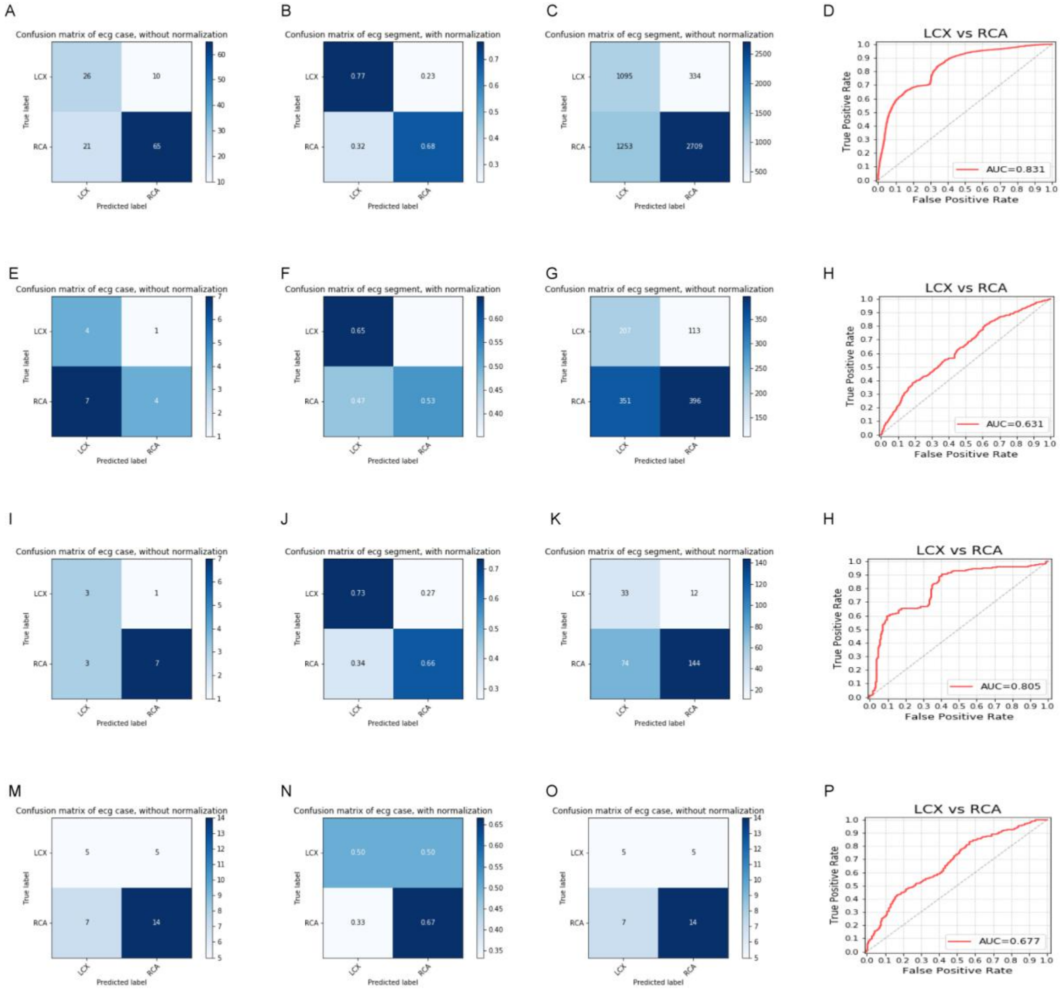

Supplementary Fig. 10 Diagnostic performance of CNN-LSTM to predict RCA and LCX. (A, B, and C) Confusion matrices (cases, proportion, and segments) for the discrimination of RCA and LCX in training dataset. (D) ROC curve of CNN-LSTM in the training dataset. (E, F, and G) Confusion matrices (cases, proportion, and segments) in the validation dataset. (H) ROC curve in the validation dataset. (I, J, and K) Confusion matrices (cases, proportion, and segments) in Test 1 dataset. (L) ROC curve in Test 1 dataset. (M, N, and O) Confusion matrices (cases, proportion, and segments) in Test 2 dataset. (P) ROC curve in Test 2 dataset.

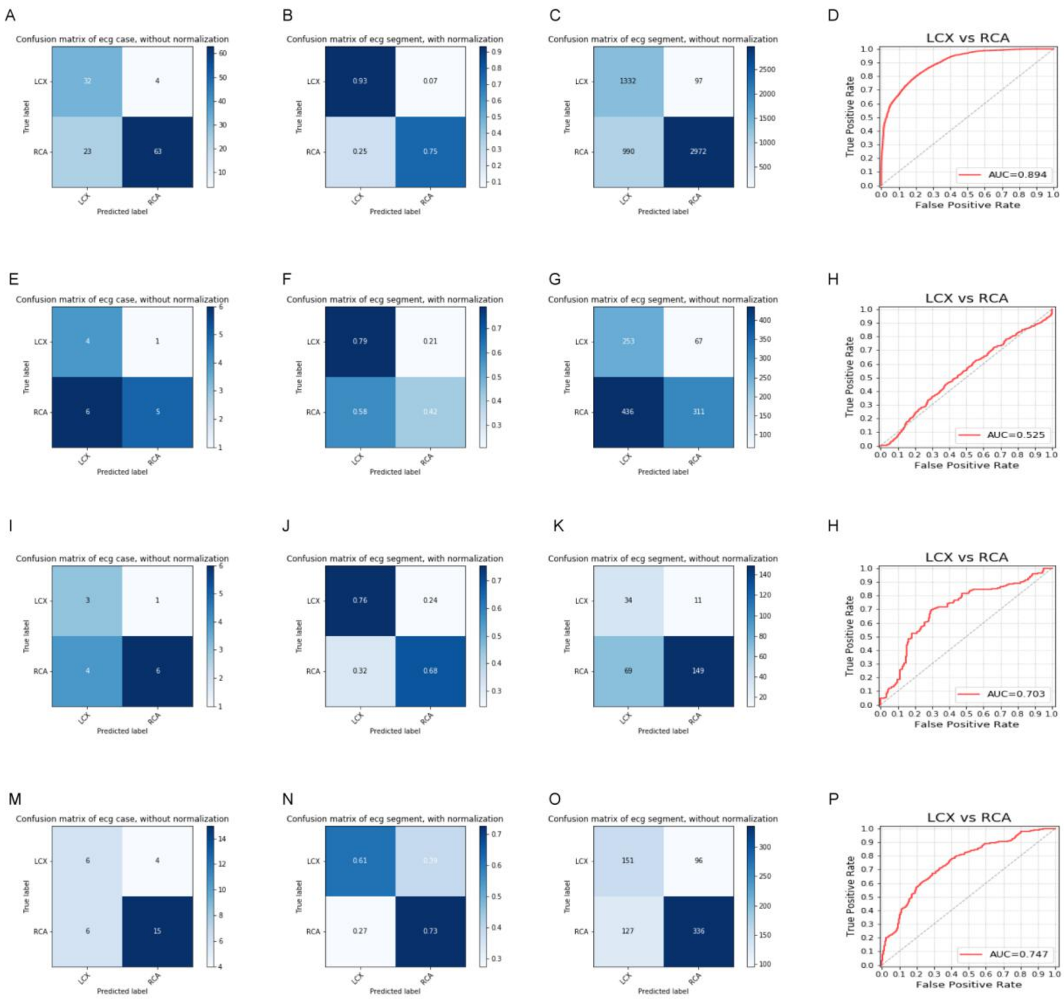

Supplementary Fig. 11 Diagnostic performance of CNN to predict RCA and LCX. (A, B, and C) Confusion matrices (cases, proportion, and segments) for the discrimination of RCA and LCX in training dataset. (D) ROC curve of CNN in the training dataset. (E, F, and G) Confusion matrices (cases, proportion, and segments) in the validation dataset. (H) ROC curve in the validation dataset. (I, J, and K) Confusion matrices (cases, proportion, and segments) in Test 1dataset. (L) ROC curve in Test 1 dataset. (M, N, and O) Confusion matrices (cases, proportion, and segments) in Test 2 dataset. (P) ROC curve in Test 2 dataset.

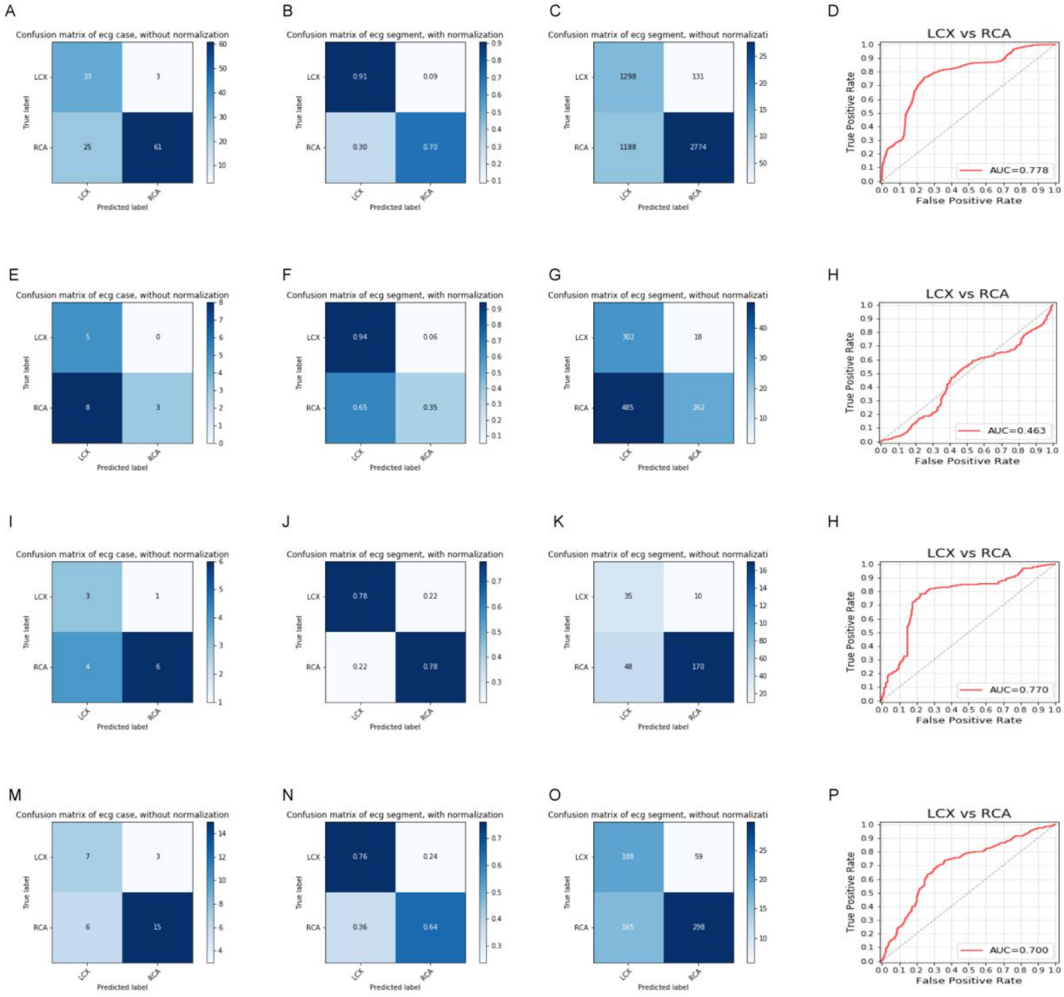

Supplementary Fig. 12 Diagnostic performance of LSTM to predict RCA and LCX. (A, B, and C) Confusion matrices (cases, proportion, and segments) for the discrimination of RCA and LCX in training dataset. (F) ROC curve of LSTM in the training dataset. (E, F, and G) Confusion matrices (cases, proportion, and segments) in the validation dataset. (H) ROC curve in the validation dataset. (I, J, and K) Confusion matrices (cases, proportion, and segments) in Test 1 dataset. (I) ROC curve in Test 1 dataset. (M, N, and O) Confusion matrices (cases, proportion, and segments) in Test 2 dataset. (P) ROC curve in Test 2 dataset.

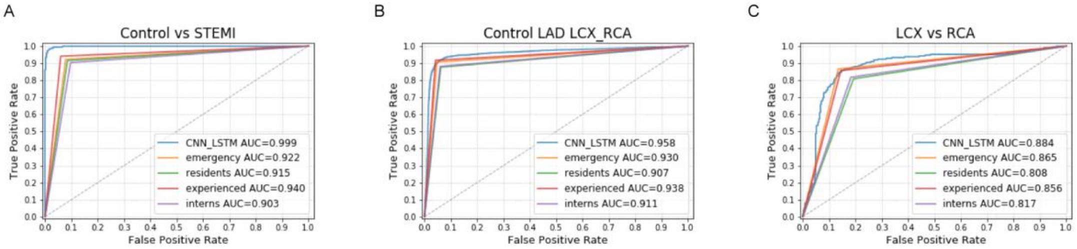

Supplementary Fig. 13 Diagnostic performance of different levels of doctors. (A) ROC curve for the discrimination of control and STEMI. (B) ROC curve for the discrimination of Control, LAD, and RCA-LCX. (C) ROC curve for the discrimination of RCA and LCX.
